# WHO European Childhood Obesity Surveillance Initiative: health-risk behaviours on nutrition and physical activity in 6–9-year-old schoolchildren

**DOI:** 10.1017/S1368980015001937

**Published:** 2015-07-01

**Authors:** Trudy MA Wijnhoven, Joop MA van Raaij, Agneta Yngve, Agneta Sjöberg, Marie Kunešová, Vesselka Duleva, Ausra Petrauskiene, Ana I Rito, João Breda

**Affiliations:** 1 Division of Noncommunicable Diseases and Promoting Health through the Life-Course, WHO Regional Office for Europe, UN City, Marmorvej 51, DK-2100 Copenhagen Ø, Denmark; 2 Centre for Nutrition, Prevention and Health Services, National Institute for Public Health and the Environment, Bilthoven, The Netherlands; 3 Division of Human Nutrition, Wageningen University, Wageningen, The Netherlands; 4 School of Hospitality, Culinary Arts and Meal Science, Örebro University, Grythyttan, Sweden; 5 Department of Food and Nutrition and Sport Science, University of Gothenburg, Gothenburg, Sweden; 6 Obesity Management Centre, Institute of Endocrinology, Prague, Czech Republic; 7 Department of Food and Nutrition, National Center of Public Health and Analyses, Sofia, Bulgaria; 8 Department of Preventive Medicine, Lithuanian University of Health Sciences, Kaunas, Lithuania; 9 National Health Institute Doutor Ricardo Jorge, Lisbon, Portugal

**Keywords:** Food consumption, Physical activity, Obesity, Schoolchildren, Europe

## Abstract

**Objective:**

To assess to what extent eight behavioural health risks related to breakfast and food consumption and five behavioural health risks related to physical activity, screen time and sleep duration are present among schoolchildren, and to examine whether health-risk behaviours are associated with obesity.

**Design:**

Cross-sectional design as part of the WHO European Childhood Obesity Surveillance Initiative (school year 2007/2008). Children’s behavioural data were reported by their parents and children’s weight and height measured by trained fieldworkers. Descriptive statistics and logistic regression analyses were performed.

**Setting:**

Primary schools in Bulgaria, Lithuania, Portugal and Sweden; paediatric clinics in the Czech Republic.

**Subjects:**

Nationally representative samples of 6–9-year-olds (*n* 15 643).

**Results:**

All thirteen risk behaviours differed statistically significantly across countries. Highest prevalence estimates of risk behaviours were observed in Bulgaria and lowest in Sweden. Not having breakfast daily and spending screen time ≥2 h/d were clearly positively associated with obesity. The same was true for eating ‘foods like pizza, French fries, hamburgers, sausages or meat pies’ >3 d/week and playing outside <1 h/d. Surprisingly, other individual unhealthy eating or less favourable physical activity behaviours showed either no or significant negative associations with obesity. A combination of multiple less favourable physical activity behaviours showed positive associations with obesity, whereas multiple unhealthy eating behaviours combined did not lead to higher odds of obesity.

**Conclusions:**

Despite a categorization based on international health recommendations, individual associations of the thirteen health-risk behaviours with obesity were not consistent, whereas presence of multiple physical activity-related risk behaviours was clearly associated with higher odds of obesity.

The determinants of childhood obesity are complex and numerous^(^ ^1^ ^)^. Mostly, it is generally accepted that individual eating and physical activity patterns as well as sedentary behaviours are important contributors^(^ ^2^ ^)^. Alongside an increase of overweight and obesity levels in children observed during the last decades at the global^(^ ^3^ ^–^ ^5^ ^)^ and European levels^(^ ^2^ ^,^ ^6^ ^)^, in many countries a shift has been observed from diets based mainly on unprocessed foods to diets high in fat, sugar and salt^(^ ^7^ ^)^. Simultaneously, trends towards decreased habitual physical activity levels and increased sedentary behaviours (such as screen time) have been observed^(^ ^7^ ^)^. Lifestyle behaviours related to diet and physical activity tend to track from childhood into adulthood^(^ ^8^ ^)^ and children with excess body weight are likely to stay overweight or obese in adulthood^(^ ^9^ ^)^. Obesity preventive measures aiming to improve diets and physical activity would thus be priority actions during childhood^(^ ^1^ ^,^ ^6^ ^)^.

Monitoring dietary patterns and physical activity levels would ideally be part and parcel of an obesity surveillance system^(^ ^10^ ^)^ and is important for evaluating policy implementation and combating the obesity epidemic^(^ ^2^ ^)^. The European Childhood Obesity Surveillance Initiative (COSI) was initiated in 2006 by the WHO Regional Office for Europe and some Member States as a follow-up to the WHO European Charter on Counteracting Obesity^(^ ^11^ ^)^. COSI is a population-based monitoring system that measures at regular intervals the levels of overweight and obesity (based on mandatory measurements of the children’s weight and height) among primary-school children aged 6–9 years in the WHO European Region. The COSI protocol also includes the administration on a voluntary basis of a family form that gathers information on children’s lifestyle behaviours such as food consumption frequency, physical activity, screen time and sleep duration, as well as on family socio-economic characteristics. Countries could choose all or just some of the questions in this family form^(^ ^12^ ^)^. The first COSI data collection round took place in school year 2007/2008 in which thirteen countries (Belgium (Flemish region only), Bulgaria, Cyprus, Czech Republic, Ireland, Italy, Latvia, Lithuania, Malta, Norway, Portugal, Slovenia and Sweden) participated. All these countries except Cyprus had a complete set of children’s data on weight and height measurements, and five countries also had a complete set of children’s data on dietary intake and physical activity indicators. The present paper describes the findings of these five countries (Bulgaria, Czech Republic, Lithuania, Portugal and Sweden) that collected data on all questions related to children’s lifestyle behaviours on the family form and provided their data to WHO according to the COSI protocol^(^ ^13^ ^)^.

The purposes of the current study were to assess to what extent behavioural health risks related to diet and physical activity are present among 6–9-year-old children in five European countries and to examine whether health-risk behaviours are associated with overweight and obesity.

## Methods

### Sampling of children

Countries applied a nationally representative school-based cluster sampling design, whereby primary schools were the primary sampling units (except the Czech Republic, where the primary sampling unit was composed of paediatric clinics). Primary schools were selected randomly from the list of all primary schools centrally available in each country through the Ministry of Education or the national school registry (or in the Czech Republic, the national list of primary-care paediatricians). Anthropometric outcome measures, such as BMI, were the initial main outcomes of interest of COSI implementation. Stratification of the primary sampling units was therefore applied if it was expected that differences in these measures across strata would be observed. This was done by the Czech Republic by region and level of urbanization and by Lithuania by district and level of urbanization. Information on the urbanization grade of the children’s residence, using the three predefined options ‘urban’, ‘semi-urban’ or ‘rural’ in all countries, was obtained. Classes formed the secondary sampling units and subsequently all children registered in the sampled classes were approached for their participation. Detailed sampling characteristics have been described elsewhere^(^ ^12^ ^,^ ^14^ ^)^.

COSI targets children aged 6, 7, 8 and 9 years old, whereby countries could choose one or more of these four age groups. If all children of the specifically targeted age group were in the same grade, then one class per school was drawn within a grade level. If the specifically targeted age group was spread across grades, however, all grades where children from this age group were present could be sampled. The majority of the thirteen countries that participated in the first data-collection round targeted one age group, including the five countries that are the subject of the present paper’s research. Bulgaria, the Czech Republic, Lithuania and Portugal targeted 7-year-olds and Sweden targeted 7- and 8-year-olds. All children in the sampled classes who were younger than 6 years or older than 9 years were excluded from the analyses (*n* 18). Moreover, data from Madeira, collected one year after the other Portuguese regions, were not included in the present Portuguese data.

### COSI family record form

The COSI family record form was based partly on the questionnaire used in the 2001/2002 round of the Health Behaviour in School-aged Children (HBSC) study that surveys 11-, 13- and 15-year-olds^(^ ^15^ ^)^. The form was given directly to the parents, sent home with the child or mailed to the child’s home, and it was often attached to the letter informing parents about the survey and asking for their consent. The form was completed by the children’s parents or caregivers, possibly together with their child. Table 1 lists the questions and their predefined answer options that were included in the form to collect data on the children’s lifestyle behaviours and on parental socio-economic status by describing their educational and occupational level, and were subject of the present paper’s research. A complete overview of all questions that were included in the COSI family record form can be found elsewhere^(^ ^12^ ^)^. For the paper’s statistical analyses, the answer options of the children’s related items were categorized into ‘healthy behaviours’ *v*. ‘health-risk behaviours’ (see also Table 1), whereby the categorization was based on international health recommendations as follows:

1. Breakfast consumption frequency: daily breakfast consumption is part of a healthy diet and contributes to the quality and quantity of a person’s daily dietary intake^(^ ^16^ ^)^ (as such categorized in the ‘healthy behaviour category’ in the present paper).
2. Food consumption frequency: eight items in the questionnaire related to food consumption frequencies were used for the analyses: (i) ‘fresh fruit’; (ii) ‘100 % fruit juice’; (iii) ‘vegetables (excluding potatoes)’; (iv) ‘soft drinks containing sugar’; (v) ‘foods like potato chips (crisps), corn chips, popcorn or peanuts’; (vi) ‘foods like candy bars or chocolate’; (vii) ‘foods like biscuits, cakes, doughnuts or pies’; and (viii) ‘foods like pizza, French fries (chips), hamburgers, sausages or meat pies’. Items (i) to (iii) are good sources of complex carbohydrates, vitamins, minerals and other substances important for good health^(^ ^17^ ^,^ ^18^ ^)^ and one of the main food groups that should contribute to a child’s daily diet^(^ ^19^ ^)^. Daily consumption of these items was considered a ‘healthy behaviour’ in the present paper. Items (iv) to (viii) tend to have a high content of saturated fats, free sugars or salt, and therefore their consumption should be limited^(^ ^20^ ^)^. Consumption of these items ≤3 d/week was considered a ‘healthy behaviour’ in the present paper.
3. Physical activity: children and adolescents aged 5–17 years should accumulate at least 60 min of moderate- to vigorous-intensity physical activity daily. Most of the daily physical activity should be aerobic. Vigorous-intensity activities should be incorporated, including those that strengthen muscle and bone, at least three times weekly. These WHO recommendations represent a minimum target for daily physical activity that allows for health enhancement and prevention of non-communicable diseases^(^ ^21^ ^)^. Active transport, playing outside for ≥1 h/d and performing sport activities for ≥2 d/week are in line with the recommended levels for this age group (as such categorized in the ‘healthy behaviour category’ in the present paper).
4. Screen time: guidelines for parents from the American Academy of Pediatrics include the limitation of total media time to no more than 1–2h/d^(^ ^22^ ^)^. Spending screen time <2h/d was considered a ‘healthy behaviour’ in the present paper.
5. Sleep duration: according to normal sleep characteristics, the usual sleep duration of pre-school children aged 4 years is 12–13 h/d and shows a steady decline with increasing age in schoolchildren aged 5 years onwards. By 10 years of age, the sleep duration is 8–10 h/d^(^ ^23^ ^)^. Sleep duration of ≥9 h/d was considered a ‘healthy behaviour’ in the present paper.

**Table 1:** Questions and their predefined answer options as included in the COSI family record form to collect data on children’s lifestyle behaviours and parental socio-economic status, and categorization of the answer options for the paper’s analyses

Questions	Answer options	Categorization of answer options for the paper’s analyses
Items on children		
Breakfast consumption frequency		
‘Over a typical or usual week, how often does your child have breakfast?’	‘Every day’; ‘most days (4–6 days)’; ‘some days (1–3 days)’; ‘never’	⇒ Every day‡=‘every day’ ⇒ <7 d/week§=‘most days (4–6 days)’, ‘some days (1–3 days)’ or ‘never’
Food consumption frequency		
‘Over a typical or usual week, how often does your child eat or drink the following kinds of foods or beverages?’	‘Every day’; ‘most days (4–6 days)’; ‘some days (1–3 days)’;’ never’	Food items (i) to (iii)||: ⇒ Every day‡=‘every day’ ⇒ <7 d/week§=‘most days (4–6 days)’, ‘some days (1–3 days)’ or ‘never’ Food items (iv) to (viii): ⇒ ≤3 d/week‡=‘some days (1–3 days)’ or ‘never’ ⇒ >3 d/week§=‘every day’ or ‘most days (4–6 days)’
(i) ‘Fresh fruit’; (ii) ‘100 % fruit juice’; (iii) ‘vegetables (excluding potatoes)’; (iv) ‘soft drinks containing sugar’; (v) ‘foods like potato chips (crisps), corn chips, popcorn or peanuts’; (vi) ‘foods like candy bars or chocolate’; (vii) ‘foods like biscuits, cakes, doughnuts or pies’; (viii) ‘foods like pizza, French fries (chips), hamburgers, sausages or meat pies’		
Physical activity		
‘How does your child usually get to and from school? Please tick one box for “Going to school” and one box for “Coming from school”’	‘S/he usually takes the school bus’; ‘s/he usually goes by public transport’; ‘s/he is usually brought by car’; ‘s/he usually rides a bicycle’; ‘s/he usually walks’; ‘other’	⇒ Active (both routes)‡=‘s/he usually rides a bicycle’ or ‘s/he usually walks’ ⇒ Inactive (both routes)§=‘s/he usually takes the school bus’, ‘s/he usually goes by public transport’, ‘s/he is usually brought by car’ or other means of inactive transport ⇒ Combined (one route ‘active’ and one route ‘inactive’)
‘Is your child a member of one or more sports or dancing clubs (e.g. football, running, hockey, swimming, tennis, basketball, gymnastics, ballet, fitness, ballroom dancing, etc.)?’	‘Yes’; ‘no’	⇒ ≥2 d/week‡=‘2 days a week’, ‘3 days a week’, ‘4 days a week’, ‘5 days a week’, ‘6 days a week’ or ‘7 days a week’ ⇒ <2 d/week§=‘0 days a week’, ‘1 day a week’ or ‘not being a member of one or more sports or dancing club(s)’
‘Over a typical or usual week, on how many days does your child go to this/these sports or dancing club(s)?’	‘0 days a week’; ‘1 day a week’; ‘2 days a week’; ‘3 days a week’; ‘4 days a week’; ‘5 days a week’; ‘6 days a week’; ‘7 days a week’	
‘In his/her free time, about how many hours per day does your child usually play outside, at home or somewhere else? Please tick one box for weekdays and one box for weekends’	‘Never’; ‘less than 1 hour per day’; ‘about 1 hour per day’; ‘about 2 hours per day’; ‘about 3 or more hours per day’	⇒ ≥1 h/d‡,¶ ⇒ <1 h/d§,¶
Screen time		
‘In his/her free time, about how many hours per day does your child usually spend using a computer for playing games (other than homework), at home or somewhere else? Please tick one box for weekdays and one box for weekends’	‘Never’; ‘less than 1 hour per day’; ‘about 1 hour per day’; ‘about 2 hours per day’; ‘about 3 or more hours per day’	⇒ <2 h/d‡,‡‡ ⇒ ≥2 h/d§,‡‡
‘In his/her free time, about how many hours per day does your child usually spend watching television (including videos), at home or somewhere else? Please tick one box for weekdays and one box for weekends’	‘Never’; ‘less than 1 hour per day’; ‘about 1 hour per day’; ‘about 2 hours per day’; ‘about 3 or more hours per day’	
Sleep duration		
‘What is your child’s usual amount of sleep each day?’	___ hours and __minutes (combining night-time sleep and naps)	⇒ ≥9 h/d‡ ⇒ <9 h/d§
Items on parents		
Education		
‘What is the highest level of education you and/or your spouse/partner have completed? Please select one answer only for each of you’	‘Primary school’; ‘secondary school’; ‘undergraduate/bachelor’s degree’; ‘master’s degree or higher’	Maximum educational level of both parents: ⇒ Low=both parents ‘primary school’ or ‘secondary school’ ⇒ High=at least one parent ‘undergraduate/bachelor’s degree’ or ‘master’s degree or higher’
Occupation		
‘Which of the following best describes your and/or your spouse’s/partner’s main work over the last 12 months? Please select one answer only for each of you’	‘Government employed’; ‘non-government employed’; ‘self-employed’; ‘student’; ‘homemaker’; ‘unemployed, able to work’; ‘unemployed, unable to work’; ‘retired’	⇒ Both unemployed=both parents ‘student’, ‘homemaker’, ‘unemployed, able to work’, ‘unemployed, unable to work’ or ‘retired’ ⇒ One or both employed=at least one parent ‘government employed’, ‘non-government employed’ or ‘self-employed’

COSI, Childhood Obesity Surveillance Initiative.

‡ ‘Healthy behaviour’ category, which served as the reference category in the logistic regression analyses.

§ ‘Health-risk behaviour’ category, which served as the exposure of interest and was used for the calculation of the three risk behaviour scores.

|| The answers for the two items ‘fresh fruit’ and ‘100 % fruit juice’ were combined into ‘every day’ (at least one of the items was categorized in ‘every day’) and ‘<7 d/week’ (both items were categorized in ‘<7 d/week’).

¶ Numerical values were assigned to the items ‘playing outside on a weekday’ and ‘playing outside on a weekend day’ enabling the conversion of this item to a numerical scale (‘never’=0; ‘less than 1 hour per day’=0·5; ‘about 1 hour per day’=1; ‘about 2 hours per day’=2; ‘about 3 or more hours per day’=3). Usual outside play time per day was calculated weighing weekday (5/7) and weekend hours (2/7) accordingly.

‡‡ Numerical values were assigned to the items ‘using a computer’ and ‘watching television’ on a weekday or a weekend day enabling the conversion of these two items to a numerical scale (‘never’=0; ‘less than 1 hour per day’=0·5; ‘about 1 hour per day’=1; ‘about 2 hours per day’=2; ‘about 3 or more hours per day’=3). Total screen time per day was calculated as the sum of the two items weighing weekday (5/7) and weekend hours (2/7) accordingly.

### Food and physical activity risk behaviour scores

A ‘food-risk behaviour score’ and a ‘physical activity-risk behaviour score’ were created for each child based on the presence of eight food-related and five physical activity-related (including screen time and sleep duration) health-risk behaviours, respectively (see Table 1). One point was assigned to the presence of each risk behaviour and subsequently all points were added together. The ‘food-risk behaviour score’ could range from 0 (none of the food-risk behaviours present) to 8 points (all food-risk behaviours present), the food-risk behaviours being:

1. having breakfast <7 d/week;
2. eating fruit <7 d/week;
3. eating vegetables (excluding potatoes) <7 d/week;
4. drinking soft drinks containing sugar >3 d/week;
5. eating foods like potato chips (crisps), corn chips, popcorn or peanuts >3 d/week;
6. eating foods like candy bars or chocolate >3 d/week;
7. eating foods like biscuits, cakes, doughnuts or pies >3 d/week; and
8. eating foods like pizza, French fries (chips), hamburgers, sausages or meat pies >3 d/week.

The ‘physical activity-risk behaviour score’ could range from 0 (none of the physical activity-risk behaviours present) to 5 points (all physical activity-risk behaviours present), the physical activity-risk behaviours being:

1. using inactive transportation going to and from school;
2. going to a sports or dancing club <2 d/week;
3. playing outside <1 h/d;
4. spending screen time ≥2 h/d; and
5. sleep duration <9 h/d.

In addition, a ‘health-risk behaviour score’ was created for each child by combining the ‘food-risk behaviour score’ and the ‘physical activity-risk behaviour score’ ranging from 0 (none of the health-risk behaviours present) to 13 points (all health-risk behaviours present). The three scores were assigned only to the children with no missing values for any of the thirteen health-risk behaviours under study.

### Weight and height measurements

Children’s weight and height were measured by fieldworkers who were trained in measuring according to WHO standardized techniques^(^ ^13^ ^,^ ^24^ ^)^. Children were asked to take off their shoes and socks, as well as all heavy clothing and to remove items such as wallets or mobile phones. The clothes worn by a child during the weight and height measurements were noted by using four predefined types of clothing: ‘underwear only’, ‘gym clothes (e.g. shorts and t-shirt only)’, ‘light clothing (e.g. t-shirt, cotton trousers or skirt)’ or ‘heavy clothing (e.g. sweater and jeans)’. Body weight was measured to the nearest 0·1 kg with portable digital (mainly manufacturer-calibrated) scales and body height was measured standing upright to the nearest 0·1 cm with portable stadiometers. Body weight was adjusted for the weight of the clothes worn, whereby the average weights of types of clothing were provided by each country. BMI was calculated using the formula: adjusted weight/height^2^ (kg/m^2^). The 2007 WHO recommended growth reference^(^ ^25^ ^)^ for school-age children was used to compute BMI-for-age *Z*-scores. Children who did not have biologically plausible values (i.e. a BMI-for-age *Z*-score between –5 and +5^(^ ^26^ ^)^) were excluded from the analyses (*n* 31). Thinness was defined as the proportion of children with a BMI-for-age value <−2 *Z*-score (BMI-for-age value below –2 sd relative to the median BMI-for-age of the 2007 WHO growth reference)^(^ ^25^ ^)^, normal weight as the proportion of children with a BMI-for-age value ≥–2 and ≤+1 *Z*-score, overweight as the proportion of children with a BMI-for-age value above +1 *Z*-score, obesity as the proportion of children with a BMI-for-age value above +2 *Z*-score and pre-obesity as the proportion of children with a BMI-for-age value >+1 and ≤+2 *Z*-score. The prevalence estimates for overweight children include those who are obese and the prevalence estimates for obese children exclude those who are pre-obese^(^ ^24^ ^)^. The three physical status categories ‘normal weight’, ‘overweight’ and ‘obese’ were used as outcome measures in the analyses.

### Statistical analyses

For each country-specific data set, the Shapiro–Francia test was used to assess whether the three calculated risk-behaviour scores were normally distributed. Preliminary analyses revealed that all country-specific food-risk behaviour and health-risk behaviour scores were skewed, and thus the final descriptive analyses included the calculation of their medians and quartiles. Mean and standard deviation were computed for continuous normally distributed variables and percentage for categorical variables. Differences in percentages and medians across the countries were examined using the *χ* ^2^ test and the Kruskal–Wallis test, respectively. If the *χ* ^2^ tests were found significant, the Marascuilo procedure^(^ ^27^ ^)^ was used for the multi-group comparisons of proportions between countries.

Bivariate multilevel logistic regression analyses were performed, and odds ratios along with 95 % confidence intervals were obtained by country to explore associations between each of the thirteen children’s health-risk behaviours individually (exposure variables) and the odds of being overweight as well as the odds of being obese (compared with normal-weight children). The association was considered positive when a health-risk behaviour was associated with higher odds of the outcome of interest (overweight or obesity), and the association was considered negative when the health-risk behaviour was associated with lower odds of the outcome.

Nine of the preliminary country-specific analyses performed, to test the interaction between the sex of the child and each of the thirteen behaviours associated with overweight or with obesity, indicated that the analyses needed to be stratified by sex. All country-specific final analyses were therefore adjusted for the children’s sex and age, and included random effects for the primary sampling units (schools in Bulgaria, Lithuania, Portugal and Sweden; paediatric clinics in the Czech Republic) to account for the clustered study design. The analyses were also done for the five countries together and included the random effects for country as well.

In the multivariable multilevel logistic regression analyses, children from the total study group with a missing value for any of the children’s health-risk behaviours (*n* 3731), children’s residential urbanization grade (*n* 77), parental education (*n* 1277) or parental occupation were excluded (*n* 1710). None of the health-risk behaviours showed multicollinearity, which was tested with Pearson correlation analyses and determined by a correlation coefficient of 0·80 or higher. All health-risk behaviours were included simultaneously in the multivariable analyses and similar adjustments were made as with the bivariate analyses. In addition, the analyses were adjusted for children’s residential urbanization grade, parental education and parental occupation because preliminary analyses suggested the presence of an association between each of these three variables individually and children’s overweight or obesity in at least one country. This subgroup of children for the multivariable analyses was also used to estimate the odds of being overweight or the odds of being obese being associated with the number of risk behaviours present relative to zero or one of the risk behaviours present. The linear trend test of odds ratios was performed using the likelihood ratio test.

A *P* value of <0·05 was used to define statistical significance. All statistical analyses were performed in the statistical software package Stata version 10·1.

## Results

### Children’s characteristics

The initial sample included 19 494 children who were present on the day of the measurements and of whom the highest number of refusals was observed in Bulgaria (13·3 %) and Sweden (11·8 %). Of the 18 183 children with complete information on age, sex and anthropometric measures, 86·3 % returned a filled out COSI family form. The subgroup of children without any missing values for the variables used for the multivariable analyses included in total 5126 fewer children (Bulgaria, 27·4 %; Czech Republic, 32·2 %; Lithuania, 29·6 %; Portugal, 39·9 %; Sweden, 35·5 %) than the total study group of 15 643 children.

Table 2 summarizes some children’s characteristics in the five countries. In the total study group, mean age ranged from 7·0 years in the Czech Republic to 8·4 years in Sweden, and boys and girls were equally represented. The prevalence of overweight ranged from 20·9 to 37·6 % and the prevalence of obesity ranged from 6·5 to 14·6 %. The prevalence figures of Czech, Lithuanian and Swedish children did not differ from each other, but their values were statistically significantly lower than those of Bulgarian and Portuguese children. The subgroup of children without missing values on any of the health-risk behaviours, children’s residential urbanization grade, parental education and parental occupation showed similar patterns (Table 2).

**Table 2:** Characteristics of the study population by country: nationally representative samples of 6–9-year-olds, WHO European Childhood Obesity Surveillance Initiative, school year 2007/2008

Characteristic	Bulgaria	Czech Republic	Lithuania	Portugal	Sweden
Children who were present on the day of the anthropometric measurements: total (*n*)	3914	1695	4955	3592	5338
Children who participated in the anthropometric measurements: total (*n*)‡	3392	1695	4948	3590	4708
Children with complete data on age, sex, weight and height measures: total (*n*)	3381	1692	4939	3590	4581
Children who returned a filled out family form: total (*n*)§	3427	1660	4436	3063	3711
Total study group of children: total (*n*)||	3267	1633	4084	3026	3633
Age (years)					
Mean	7·7	7·0	7·8	7·5	8·4
sd	0·3	0·3	0·3	0·6	0·6
Boys					
*n*	1619	815	2064	1509	1874
%	49·6	49·9	50·5	49·9	51·6
Prevalence of overweight (%)¶	28·8^b^	20·9^c^	23·1^c^	37·6^a^	23·2^c^
Prevalence of obesity (%)¶	12·4^a^	7·3^b^	8·3^b^	14·6^a^	6·5^b^
Prevalence of normal weight (%)¶	68·4^b^	76·3^a^	74·9^a^	61·5^c^	75·6^a^
Prevalence of thinness (%)¶	2·9^a^	2·8^a^	2·0^a^	0·9^b^	1·1^b^
BMI-for-age *Z*-score					
Mean	0·35	0·13	0·27	0·71	0·27
sd	1·36	1·21	1·18	1·20	1·07
Subgroup of children: total (*n*)‡‡	2373	1107	2874	1818	2345
Age (years)					
Mean	7·7	7·0	7·8	7·5	8·4
sd	0·3	0·3	0·3	0·6	0·6
Boys					
*n*	1189	543	1475	901	1247
%	50·1	49·1	51·3	49·6	53·2
Prevalence of overweight (%)¶	29·3^b^	20·1^c^	24·5^c^	37·7^a^	22·6^c^
Prevalence of obesity (%)¶	12·6^a^	7·4^b,c^	8·9^b^	13·8^a^	5·8^c^
Prevalence of normal weight (%)¶	68·1^b^	77·2^a^	73·5^a^	61·6^c^	76·1^a^
Prevalence of thinness (%)¶	2·6^a^	2·7^a,b^	2·0^a,b^	0·7^c^	1·3^b,c^
BMI-for-age *Z*-score					
Mean	0·38	0·13	0·30	0·70	0·25
sd	1·36	1·22	1·20	1·19	1·07

^a,b,c^Proportions within a row with unlike superscript letters were significantly different (Marascuilo procedure). Superscripts are ranked by decreasing prevalence, whereby the highest prevalence was indicated with superscript a.

‡ All children who agreed to have their weight and height measured, including children with missing information on age or sex.

§ Out of the children who returned a filled out family form, 3285 Bulgarian, 1649 Czech, 4089 Lithuanian, 3032 Portuguese and 3637 Swedish children had complete information on age, sex, weight and height measures.

|| Children with complete information on sex, whose age was between 6 and 9 years old, whose weight and height were measured, whose BMI-for-age *Z*-score was within the normal range (≥–5 and ≤+5) and who returned a filled out family record form.

¶ Overweight is defined as the proportion of children with a BMI-for-age value >+1 *Z*-score (i.e. BMI-for-age above +1 sd relative to the median BMI-for-age of the 2007 WHO growth reference)^(^ ^25^ ^)^, obesity as the proportion of children with a BMI-for-age value >+2 *Z*-score, normal weight as the proportion of children with a BMI-for-age value ≥–2 and ≤+1 *Z*-score, and thinness as the proportion of children with a BMI-for-age value <–2 *Z*-score. Statistically significant difference in proportions across the countries (*χ* ^2^ test; *P*<0·001).

‡‡ All criteria for the total study group of children, as well as no missing values for the thirteen health-risk behaviours, children’s residential urbanization grade, parental education and parental occupation.

Outcomes on the educational level and the main occupation over the last 12 months of the children’s parents as well as the children’s residential urbanization grade are given in Supplementary Table 1 (see online supplementary material). In summary, the percentage of parents with either primary school or secondary school as their highest completed educational level was 37·9 % in Lithuania, 49·3 % in Sweden, 62·0 % in the Czech Republic, 63·2 % in Bulgaria and 80·2 % in Portugal. Unemployment of both parents varied between 2·0 and 3·6 % in the Czech Republic, Lithuania, Portugal and Sweden and was 10·4 % in Bulgaria. The children’s residential area was mainly urban in Bulgaria (78·8 %), Portugal (66·4 %) and in the Czech Republic (47·8 %), mainly semi-urban in Lithuania (40·9 %) and mainly rural in Sweden (55·3 %).

### Breakfast and food consumption frequencies

Supplementary Table 2 (see online supplementary material) presents the proportion of consumption frequencies of breakfast and eight food items over a usual week for each answer category in the five countries. The first part of Table 3 is derived from Supplementary Table 2 and presents the frequencies of food-related health-risk behaviours. As shown in Table 3, the prevalence of all food-related risk behaviours differed statistically significantly across the countries. Less favourable food behaviours (shown by lower-ranked superscripts, e.g. a and b) were mainly found in Bulgaria and more favourable food behaviours in Sweden (shown by higher-ranked superscripts, e.g. d and e). Almost 40 % or more of the Bulgarian children ate foods like ‘pizza, French fries (chips), hamburgers, sausages or meat pies’, ‘biscuits, cakes, doughnuts or pies’, ‘potato chips (crisps), corn chips, popcorn or peanuts’ or ‘candy bars or chocolate’ on >3 d/week, while 6 % or fewer of the Swedish children did so. The percentage of children who did not have breakfast every day ranged from 4·4 % in Portugal to 32·5 % in Lithuania.

**Table 3:** Prevalence (%) of children’s health-risk behaviours in the total study group‡ by country: nationally representative samples of 6–9-year-olds, WHO European Childhood Obesity Surveillance Initiative, school year 2007/2008

Health-risk behaviour		Bulgaria	Czech Republic	Lithuania	Portugal	Sweden
Breakfast and food consumption frequency						
1.	Having breakfast <7 d/week§	21·1^b^	24·3^b^	32·5^a^	4·4^c^	5·7^c^
2.	Eating fruit|| <7 d/week§	64·3^a^	46·8^b^	62·8^a^	35·0^c^	31·2^d^
3.	Eating vegetables (excluding potatoes) <7 d/week§	76·4^a^	71·7^b^	76·8^a^	60·6^c^	46·6^d^
4.	Drinking soft drinks containing sugar >3 d/week§	38·6^b^	46·5^a^	19·5^c^	20·2^c^	8·5^d^
5.	Eating foods like potato chips (crisps), corn chips, popcorn or peanuts >3 d/week§	46·5^a^	4·3^c^	9·7^b^	5·5^c^	1·1^d^
6.	Eating foods like candy bars or chocolate >3 d/week§	63·4^a^	24·4^c^	44·0^b^	11·7^d^	2·2^e^
7.	Eating foods like biscuits, cakes, doughnuts or pies >3 d/week§	46·4^a^	24·2^c^	33·3^b^	25·8^c^	6·4^d^
8.	Eating foods like pizza, French fries (chips), hamburgers, sausages or meat pies >3 d/week§	39·4^a^	2·2^c^	7·3^b^	6·6^b^	2·0^c^
Physical activity						
9.	Using inactive transportation going to and from school§	29·3^d^	38·8^c^	39·2^c^	66·9^a^	47·8^b^
10.	Going to a sports or dancing club <2 d/week§	79·0^a^	64·3^c^	69·4^b^	70·5^b^	48·8^d^
11.	Playing outside <1 h/d§	4·8^c^	5·2^c^	7·5^b^	35·0^a^	7·7^b^
Screen time and sleep duration						
12.	Spending screen time ≥2 h/d§	70·3^b^	32·6^d^	74·2^a^	34·7^d^	39·5^c^
13.	Sleep duration <9 h/d§	19·1^a^	5·6^c^	11·9^b^	13·1^b^	3·5^d^

^a,b,c,d,e^Within each health-risk behaviour item, proportions with unlike superscript letters were significantly different (Marascuilo procedure). Superscripts are ranked by decreasing prevalence, whereby the highest prevalence was indicated with superscript a.

‡ Children with complete information on sex, whose age was between 6 and 9 years old, whose weight and height were measured, whose BMI-for-age *Z*-score was within the normal range (≥–5 and ≤+5) and who returned a filled out family record form.

§ Statistically significant difference in proportions across the countries (*χ* ^2^ test; *P*<0·001).

|| Combination of ‘fresh fruit’ and ‘100 % fruit juice’.

### Physical activity, screen time and sleep duration

Supplementary Table 3 (see online supplementary material) presents the proportion of items related to physical activity and screen time for each answer category in the five countries. The second part of Table 3 is derived from Supplementary Table 3 and presents the frequencies of physical activity-related health-risk behaviours. As shown in Table 3, the prevalence of all physical activity-related risk behaviours differed statistically significantly across the countries. Less favourable physical activity-related behaviours were found in Bulgaria and Portugal and more favourable physical activity-related behaviours mainly in the Czech Republic. The majority of the Bulgarian (79·0 %) and Portuguese (70·5 %) children did not go to a sports or dancing club at all or only once weekly, and 35 % of the Portuguese children did not play outside for ≥1 h/d. More than 70 % of the Bulgarian and Lithuanian children participated in screen time ≥2 h/d, whereas the percentage in the other three countries ranged between 30 and 40 %.

### Food and physical activity risk-behaviour scores

A food-risk behaviour score of maximum 8 (not favourable on any of the behaviours) was found in 0·2 % of the total subgroup of children (*n* 10 517) and a physical activity-risk behaviour score of maximum 5 in 0·3 %. A food-risk behaviour score of 0 (favourable on all behaviours) was found in 14·3 % of the total subgroup of children and a physical activity-risk behaviour score of 0 in 7·9 %. None of the children had the maximum health-risk behaviour score of 13 and 2·2 % of the children had a health-risk behaviour score of 0. Table 4 displays the median values of the three calculated risk-behaviour scores (based on the presence of food-related and physical activity-related health-risk behaviours). On average, Bulgarian children had the highest food-risk and health-risk behaviour scores and Swedish children the lowest.

**Table 4:** Median values of the ‘food-risk behaviour score’, ‘physical activity-risk behaviour score’ and ‘health-risk behaviour score’, in a subgroup of children without missing data‡, by country: nationally representative samples of 6–9-year-olds, WHO European Childhood Obesity Surveillance Initiative, school year 2007/2008

	Bulgaria		Czech Republic		Lithuania		Portugal		Sweden		Total five countries	
Score	Median	Q1–Q3	Median	Q1–Q3	Median	Q1–Q3	Median	Q1–Q3	Median	Q1–Q3	Median	Q1–Q3
Food-risk behaviour score§,||	4	3–5	2	1–3	3	2–4	1	1–2	1	0–2	2	1–3
Physical activity-risk score¶,||	2	2–3	1	1–2	2	1–3	2	1–3	1	1–2	2	1–2
Health-risk behaviour score‡‡,||	6	4–7	4	3–5	5	4–6	4	3–5	2	1–3	4	3–6

Q1, first quartile; Q3, third quartile.

‡ Children with complete information on sex, whose age was between 6 and 9 years old, whose weight and height were measured, whose BMI-for-age *Z*-score was within the normal range (≥–5 and ≤+5), who returned a filled out family record form and who had no missing values on the thirteen health-risk behaviours, children’s residential urbanization grade, parental education and parental occupation.

§ Possible range: 0–8.

|| Statistically significant difference in median scores across the countries (Kruskal–Wallis test; *P*=0·0001).

¶ Possible range: 0–5.

‡‡ Possible range: 0–13.

### Associations with obesity and overweight

For the group of children in the five countries, statistically significant bivariate associations (with adjustment for children’s sex and age) with obesity were found in four food-related risk behaviours (Table 5), whereas significant multivariable associations (with adjustment for children’s sex and age, all thirteen health-risk behaviours, children’s residential urbanization grade, parental education and parental occupation) were seen in five food-related risk behaviours (Table 6). Children were more likely to be obese when they did not have breakfast every day or ate ‘foods like pizza, French fries (chips), hamburgers, sausages or meat pies’ >3 d/week, while children were less likely to be obese when they did not eat fruit every day or ate ‘foods like potato chips (crisps), corn chips, popcorn or peanuts’ or ‘foods like biscuits, cakes, doughnuts or pies’ >3 d/week (Table 6). Playing outside <1 h/d and spending screen time ≥2 h/d were the only physical activity-related risk behaviours that were statistically significantly associated with higher odds of obesity in the total group or in a country (in both bivariate and multivariable models).

**Table 5:** Bivariate associations‡ between thirteen health-risk behaviours and obesity in the total study group§, by country: nationally representative samples of 6–9-year-olds, WHO European Childhood Obesity Surveillance Initiative, school year 2007/2008

		Bulgaria		Czech Republic		Lithuania		Portugal		Sweden		Total five countries	
Health-risk behaviour		OR	95 % CI	OR	95 % CI	OR	95 % CI	OR	95 % CI	OR	95 % CI	OR	95 % CI
Breakfast and food consumption frequency													
1.||	Having breakfast <7 d/week	**1**·33*	**1**·**03, 1**·**72**	**2**·07***	**1**·**37, 3**·**10**	**1**·71***	**1**·**35, 2**·**16**	**1**·64*	**1**·**03, 2**·**62**	1·40	0·83, 2·37	**1**·57***	**1**·**36, 1**·**82**
2.||	Eating fruit¶ <7 d/week	0·85	0·68, 1·06	1·23	0·84, 1·80	0·85	0·67, 1·07	0·86	0·69, 1·08	0·95	0·71, 1·28	0·90	0·80, 1·01
3.||	Eating vegetables (excluding potatoes) <7 d/week	**0**·77*	**0**·**59, 0**·**99**	1·48	0·93, 2·34	1·07	0·81, 1·41	0·99	0·79, 1·23	1·11	0·84, 1·46	1·00	0·89, 1·13
4.||	Drinking soft drinks containing sugar >3 d/week	0·81	0·64, 1·02	0·95	0·65, 1·40	0·99	0·74, 1·32	1·28	0·98, 1·66	1·31	0·85, 2·03	1·00	0·87, 1·14
5.||	Eating foods like potato chips (crisps), corn chips, popcorn or peanuts >3 d/week	**0**·67***	**0**·**53, 0**·**84**	1·28	0·53, 3·06	**0**·64*	**0**·**41, 0**·**99**	1·07	0·68, 1·70	–		**0**·72***	**0**·**60, 0**·**86**
6.||	Eating foods like candy bars or chocolate >3 d/week	0·91	0·72, 1·14	0·72	0·45, 1·15	0·79	0·63, 1·01	0·96	0·68, 1·35	0·34	0·08, 1·41	**0**·85*	**0**·**74, 0**·**97**
7.||	Eating foods like biscuits, cakes, doughnuts or pies >3 d/week	**0**·65***	**0**·**52, 0**·**81**	0·61	0·37, 1·00	0·79	0·61, 1·01	0·84	0·65, 1·08	0·71	0·39, 1·31	**0**·74***	**0**·**64, 0**·**84**
8.||	Eating foods like pizza, French fries (chips), hamburgers, sausages or meat pies >3 d/week	0·89	0·71, 1·12	2·25	0·83, 6·04	1·16	0·77, 1·76	0·92	0·59, 1·43	1·45	0·63, 3·33	0·97	0·82, 1·17
Physical activity													
9.||	Using inactive transportation going to and from school	1·10	0·86, 1·42	0·71	0·46, 1·08	1·26	0·97, 1·62	0·91	0·70, 1·19	1·19	0·87, 1·63	1·07	0·94, 1·22
10.||	Going to a sports or dancing club <2 d/week	0·81	0·62, 1·06	1·06	0·70, 1·60	**1**·35*	**1**·**03, 1**·**76**	1·12	0·88, 1·43	1·17	0·88, 1·56	1·09	0·96, 1·23
11.||	Playing outside <1 h/d	**1**·65*	**1**·**05, 2**·**60**	0·71	0·28, 1·80	**1**·50*	**1**·**02, 2**·**20**	1·04	0·83, 1·31	1·51	0·94, 2·45	**1**·21*	**1**·**03, 1**·**44**
Screen time and sleep duration													
12.||	Spending screen time ≥2 h/d	1·16	0·90, 1·50	**1**·64*	**1**·**10, 2**·**46**	**1**·66***	**1**·**24, 2**·**24**	1·24	0·98, 1·57	**1**·73***	**1**·**31, 2**·**29**	**1**·43***	**1**·**26, 1**·**62**
13.||	Sleep duration <9 h/d	1·03	0·78, 1·36	0·79	0·31, 2·01	0·90	0·62, 1·30	**1**·38*	**1**·**02, 1**·**85**	**2**·32**	**1**·**28, 4**·**22**	1·15	0·97, 1·36

–, no observations for obese children. Significance levels: *P<0·05, **P≤0·01, ***P≤0·001; significant associations are shown in bold font.

‡ All bivariate analyses were adjusted for the children’s sex and age and included random effects for the primary sampling units. The analyses for the five countries together also included random effects for country.

§ Normal-weight or obese children with complete information on sex, whose age was between 6 and 9 years old, whose weight and height were measured, whose BMI-for-age *Z*-score was within the normal range (≥–5 and ≤+5) and who returned a filled out family record form. Obesity is defined as the proportion of children with a BMI-for-age value >+2 *Z*-score (i.e. BMI-for-age above +2 sd relative to the median BMI-for-age of the 2007 WHO growth reference)^(^ ^25^ ^)^ and was compared against normal-weight children (BMI-for-age value ≥–2 and ≤+1 *Z*-score).

|| Reference categories for each health-risk behaviour were: (i) having breakfast every day; (ii) eating fruit every day; (iii) eating vegetables (excluding potatoes) every day; (iv) drinking soft drinks containing sugar ≤3 d/week; (v) eating foods like potato chips (crisps), corn chips, popcorn or peanuts ≤3 d/week; (vi) eating foods like candy bars or chocolate ≤3 d/week; (vii) eating foods like biscuits, cakes, doughnuts or pies ≤3 d/week; (viii) eating foods like pizza, French fries (chips), hamburgers, sausages or meat pies ≤3 d/week; (ix) using active transportation going to and from school; (x) going to a sports or dancing club ≥2 d/week; (xi) playing outside ≥1 h/d, (xii) spending screen time <2 h/d; and (xiii) sleep duration ≥9 h/d.

¶ Combination of ‘fresh fruit’ and ‘100 % fruit juice’.

**Table 6:** Multivariable associations‡ between thirteen health-risk behaviours and obesity in a subgroup of children without missing data§, by country: nationally representative samples of 6–9-year-olds, WHO European Childhood Obesity Surveillance Initiative, school year 2007/2008

		Bulgaria (*n* 1914)		Czech Republic (*n* 936)		Lithuania (*n* 2369)		Portugal (*n* 1371)		Sweden (*n* 1922)		Total five countries (*n* 8512)	
Health-risk behaviour		OR	95 % CI	OR	95 % CI	OR	95 % CI	OR	95 % CI	OR	95 % CI	OR	95 % CI
Breakfast and food consumption frequency													
1.||	Having breakfast <7 d/week	**1**·70***	**1**·**26, 2**·**30**	**1**·91*	**1**·**14, 3**·**21**	**1**·80***	**1**·**36, 2**·**38**	**1**·88*	**1**·**02, 3**·**46**	**2**·20*	**1**·**12, 4**·**33**	**1**·81***	**1**·**52, 2**·**15**
2.||	Eating fruit¶ <7 d/week	0·78	0·59, 1·03	0·99	0·59, 1·66	**0**·72*	**0**·**54, 0**·**96**	0·82	0·61, 1·11	0·88	0·58, 1·33	**0**·80**	**0**·**69, 0**·**93**
3.||	Eating vegetables (excluding potatoes) <7 d/week	0·83	0·60, 1·14	1·31	0·71, 2·43	1·06	0·74, 1·50	1·01	0·74, 1·36	1·14	0·78, 1·68	1·03	0·88, 1·21
4.||	Drinking soft drinks containing sugar >3 d/week	1·04	0·78, 1·39	0·78	0·48, 1·28	1·17	0·82, 1·69	1·35	0·94, 1·93	1·50	0·85, 2·66	1·09	0·92, 1·30
5.||	Eating foods like potato chips (crisps), corn chips, popcorn or peanuts >3 d/week	**0**·66**	**0**·**49, 0**·**89**	1·23	0·36, 4·21	**0**·52*	**0**·**27, 0**·**98**	1·71	0·75, 3·91	–		**0**·66***	**0**·**52, 0**·**85**
6.||	Eating foods like candy bars or chocolate >3 d/week	1·10	0·83, 1·47	0·68	0·36, 1·28	0·85	0·63, 1·14	0·59	0·33, 1·04	–		0·89	0·74, 1·06
7.||	Eating foods like biscuits, cakes, doughnuts or pies >3 d/week	**0**·69*	**0**·**51, 0**·**94**	0·69	0·36, 1·31	0·73	0·53, 1·02	0·79	0·56, 1·14	0·73	0·34, 1·57	**0**·74***	**0**·**62, 0**·**88**
8.||	Eating foods like pizza, French fries (chips), hamburgers, sausages or meat pies >3 d/week	1·23	0·90, 1·68	2·24	0·56, 8·89	**1**·89*	**1**·**09, 3**·**31**	1·02	0·45, 2·27	1·53	0·52, 4·48	**1**·32*	**1**·**04, 1**·**69**
Physical activity													
9.||	Using inactive transportation going to and from school	1·11	0·82, 1·49	0·71	0·42, 1·20	1·16	0·86, 1·58	1·01	0·71, 1·43	1·32	0·89, 1·96	1·12	0·96, 1·30
10.||	Going to a sports or dancing club <2 d/week	0·98	0·72, 1·33	0·88	0·53, 1·47	1·29	0·95, 1·76	1·16	0·84, 1·60	1·02	0·70, 1·49	1·07	0·92, 1·25
11.||	Playing outside <1 h/d	1·37	0·80, 2·37	0·63	0·19, 2·16	**1**·59*	**1**·**03, 2**·**45**	1·02	0·76, 1·37	1·28	0·64, 2·59	1·18	0·95, 1·45
Screen time and sleep duration													
12.||	Spending screen time ≥2 h/d	1·26	0·94, 1·69	**1**·71*	**1**·**04, 2**·**80**	**1**·81***	**1**·**27, 2**·**59**	1·30	0·96, 1·75	**1**·83***	**1**·**26, 2**·**65**	**1**·55***	**1**·**33, 1**·**81**
13.||	Sleep duration <9 h/d	1·16	0·84, 1·59	0·77	0·26, 2·25	0·87	0·56, 1·33	0·96	0·63, 1·46	2·17	0·98, 4·78	1·07	0·87, 1·31

–, no observations for obese children and thus this characteristic was excluded from the Swedish multivariable analyses. Significance levels: * *P*<0·05, ** *P*≤0·01, *** *P*≤0·001; significant associations are shown in bold font.

‡ All multivariable analyses were adjusted for the children’s sex and age, included all thirteen health-risk behaviours simultaneously (except in the Swedish analyses), as well as children’s residential urbanization grade, parental education and parental occupation and included random effects for the primary sampling units. The analyses for the five countries together also included random effects for country.

§ Normal-weight or obese children with complete information on sex, whose age was between 6 and 9 years old, whose weight and height were measured, whose BMI-for-age *Z*-score was within the normal range (≥–5 and ≤+5), who returned a filled out family record form and who had no missing values on any of the thirteen health-risk behaviours, children’s residential urbanization grade, parental education and parental occupation. Obesity is defined as the proportion of children with a BMI-for-age value >+2 *Z*-score (i.e. BMI-for-age above +2 sd relative to the median BMI-for-age of the 2007 WHO growth reference)^(^ ^25^ ^)^ and was compared against normal-weight children (BMI-for-age value ≥–2 and ≤+1 *Z*-score).

|| Reference categories for each health-risk behaviour were: (i) having breakfast every day; (ii) eating fruit every day; (iii) eating vegetables (excluding potatoes) every day; (iv) drinking soft drinks containing sugar ≤3 d/week; (v) eating foods like potato chips (crisps), corn chips, popcorn or peanuts ≤3 d/week; (vi) eating foods like candy bars or chocolate ≤3 d/week; (vii) eating foods like biscuits, cakes, doughnuts or pies ≤3 d/week; (viii) eating foods like pizza, French fries (chips), hamburgers, sausages or meat pies ≤3 d/week; (ix) using active transportation going to and from school; (x) going to a sports or dancing club ≥2 d/week; (xi) playing outside ≥1 h/d; (xii) spending screen time <2 h/d; and (xiii) sleep duration ≥9 h/d.

¶ Combination of ‘fresh fruit’ and ‘100 % fruit juice’.

For the group of children in the five countries, the bivariate analyses for overweight indicated statistically significant associations between the same four food-related risk behaviours and overweight as well as between spending screen time ≥2 h/d and overweight (Supplementary Table 4, see online supplementary material) as were found with the bivariate analyses for obesity (Table 5). The physical activity-related risk behaviour playing outside for <1 h/d was not associated with overweight in both the bivariate and the multivariable analyses. Furthermore, eating ‘foods like pizza, French fries (chips), hamburgers, sausages or meat pies’ on >3 d/week did not lead to statistically significant higher odds of overweight (Supplementary Table 5, see online supplementary material), as was shown with the multivariate analyses for obesity (Table 6).

Table 7 presents the associations between the three calculated risk-behaviour scores and obesity. For the group of children in the five countries, none of the food-risk behaviour scores showed a statistically significant association with obesity. A country-specific statistically significant positive association between the food-risk behaviour score of 1 and obesity was only found in Sweden, whereas the other scores did not reach the statistical significance level. The physical activity-risk behaviour score showed positive associations with obesity at the total group level, whereby children with a score of 2 to 4 were more likely to be obese than children with a score of 0. Country-specific positive associations between the physical activity-risk behaviour score and obesity also reached the statistical significance level in Lithuania and Sweden. Compared with children with a combined health-risk behaviour score of 0 or 1, the higher the score the more likely children were obese in most categories (both in the total group and in Sweden).

**Table 7:** Associations‡ between three risk behaviour scores and obesity in a subgroup of children without missing data§, by country: nationally representative samples of 6–9-year-olds, WHO European Childhood Obesity Surveillance Initiative, school year 2007/2008

	Bulgaria			Czech Republic			Lithuania			Portugal			Sweden			Total five countries		
Score category	*n*	OR	95 % CI	*n*	OR	95 % CI	*n*	OR	95 % CI	*n*	OR	95 % CI	*n*	OR	95 % CI	*n*	OR	95 % CI
Food-risk behaviour score																		
0	52	1·00		64	1·00		85	1·00		248	1·00		756	1·00		1205	1·00	
1	114	0·80	0·34, 1·86	178	0·75	0·27, 2·07	292	0·93	0·43, 2·00	449	0·99	0·66, 1·50	620	1·75*	**1**·**13, 2**·**72**	1653	1·20	0·93, 1·55
2	288	0·87	0·41, 1·83	270	0·93	0·36, 2·40	683	0·81	0·40, 1·67	407	1·05	0·69, 1·60	399	1·61	0·98¸ 2·63	2047	1·21	0·93, 1·56
3	359	0·92	0·44, 1·92	231	0·69	0·26, 1·87	671	1·05	0·52, 2·15	155	1·10	0·65, 1·85	106	1·68	0·79, 3·56	1522	1·28	0·97, 1·69
4	356	0·68	0·32, 1·44	128	0·72	0·25, 2·14	350	0·78	0·36, 1·68	68	0·82	0·39, 1·71	28	1·45	0·32, 6·50	930	0·98	0·72, 1·35
5	355	0·81	0·38, 1·70	45	0·70	0·18, 2·75	197	0·93	0·41, 2·12	27	0·76	0·25, 2·33	6	–		630	1·10	0·78, 1·55
6	253	0·65	0·30, 1·44	15	2·21	0·47, 10·53	69	1·41	0·55, 3·63	14	1·18	0·31, 4·51	4	–		355	1·04	0·70, 1·56
7	117	0·61	0·25, 1·48	4	2·07	0·16, 27·49	22	0·33	0·04, 2·76	3	2·00	0·17, 23·69	2	–		148	0·80	0·45, 1·43
8	20	0·46	0·09, 2·38	1	–		0	–		0	–		1	–		22	0·95	0·27, 3·39
Physical activity-risk behaviour score																		
0	70	1·00		130	1·00		102	1·00†		44	1·00		351	1·00††		697	1·00††	
1	411	0·70	0·36, 1·36	394	1·07	0·49, 2·34	553	1·74	0·67, 4·53	304	0·67	0·31, 1·45	742	1·79	0·90, 3·54	2404	1·21	0·87, 1·67
2	948	0·77	0·41, 1·45	306	1·67	0·77, 3·63	1009	2·39	0·94, 6·05	516	0·62	0·29, 1·31	606	2·75**	**1**·**40, 5**·**41**	3385	1·47*	**1**·**07, 2**·**03**
3	414	0·98	0·51, 1·88	92	0·98	0·35, 2·78	615	3·02*	**1**·**18, 7**·**74**	370	0·82	0·38, 1·76	206	3·03**	**1**·**40, 6**·**57**	1697	1·79***	**1**·**28, 2**·**50**
4	64	1·05	0·44, 2·49	14	–		79	2·17	0·67, 7·01	125	0·95	0·41, 2·18	17	7·19**	**1**·**72, 30**·**02**	299	1·83**	**1**·**19, 2**·**84**
5	7	–		0	–		11	4·30	0·70, 26·29	12	0·56	0·10, 3·05	0	–		30	1·26	0·41, 3·85
Health-risk behaviour score||																		
0–1	31	1·00		66	1·00		46	1·00		103	1·00		565	1·00††		811	1·00	
2	81	0·88	0·30, 2·59	136	0·62	0·20, 1·89	163	1·32	0·36, 4·84	205	0·86	0·45, 1·66	510	1·16	0·65, 2·08	1095	1·11	0·78, 1·58
3	138	1·04	0·38, 2·84	215	0·82	0·30, 2·22	338	1·75	0·51, 5·98	320	1·05	0·58, 1·92	464	2·34**	**1**·**38, 3**·**96**	1475	1·64**	**1**·**18, 2**·**27**
4	247	0·84	0·32, 2·21	208	1·14	0·43, 3·00	502	1·49	0·44, 5·05	309	1·23	0·68, 2·23	238	2·66***	**1**·**47, 4**·**81**	1504	1·64**	**1**·**17, 2**·**29**
5	316	1·00	0·39, 2·61	153	0·89	0·32, 2·52	509	1·97	0·59, 6·63	219	1·13	0·60, 2·11	98	1·26	0·49, 3·23	1295	1·68**	**1**·**19, 2**·**37**
6	297	0·70	0·26, 1·85	97	0·62	0·19, 2·02	419	1·65	0·49, 5·62	107	1·66	0·83, 3·33	33	3·69*	**1**·**26, 10**·**82**	953	**1**·50*	**1**·**04, 2**·**16**
7	342	0·75	0·29, 1·97	35	1·42	0·38, 5·29	232	1·99	0·57, 6·93	72	0·98	0·44, 2·19	8	3·58	0·40, 31·65	689	1·48*	**1**·**01, 2**·**18**
8	263	0·98	0·37, 2·59	18	0·64	0·07, 5·88	115	1·82	0·49, 6·82	23	0·73	0·19, 2·75	4	–		423	**1**·57*	**1**·**03, 2**·**41**
9	144	0·58	0·20, 1·68	5	1·64	0·14, 18·83	32	3·47	0·78, 15·46	10	–		1	–		192	1·13	0·64, 1·97
10	48	1·08	0·33, 3·52	2	–		10	–		2	–		1	–		63	1·40	0·64, 3·10
11	6	0·72	0·07, 7·67	1	–		3	–		1	–		0	–		11	7·10**	**2**·**00, 25**·**20**
12	1	–		0	–		0	–		0	–		0	–		1	–	
13	0	–		0	–		0	–		0	–		0	–		0	–	

–, sample size was 0 or none of the children in this score category were obese and thus the OR could not be estimated for this category. Significance levels of OR: *P<0·05, **P≤0·01, ***P≤0·001; significant associations are shown in bold font. Significant linear trend of OR for the respective risk-behaviour score (likelihood ratio test): †*P* <0·01, ††*P*<0·001.

‡ All analyses were adjusted for the children’s sex and age, children’s residential urbanization grade, parental education and parental occupation and included random effects for the primary sampling units. The analyses for the five countries together also included random effects for country.

§ Normal-weight or obese children with complete information on sex, whose age was between 6 and 9 years old, whose weight and height were measured, whose BMI-for-age *Z*-score was within the normal range (≥–5 and ≤+5), who returned a filled out family record form and who had no missing values on any of the thirteen health-risk behaviours, children’s residential urbanization grade, parental education and parental occupation. Obesity is defined as the proportion of children with a BMI-for-age value >+2 *Z*-score (i.e. BMI-for-age above +2 sd relative to the median BMI-for-age of the 2007 WHO growth reference)^(^ ^25^ ^)^ and was compared against normal-weight children (BMI-for-age value ≥–2 and ≤+1 *Z*-score).

|| The reference category was not set as a health risk score of 0 but 0–1, because only six Bulgarian, fifteen Czech, seven Lithuanian, nine Portuguese and 160 Swedish children obtained a health risk score of 0.

Supplementary Table 6 (see online supplementary material) shows the associations between the three risk-behaviour scores and overweight. Same as with obesity (Table 7), none of the food-risk behaviour scores were associated with overweight. The presence of two to four physical activity-related risk behaviours was clearly associated with higher odds of overweight, whereas the combination of four food- and physical activity-related risk behaviours only was positively associated with overweight.

## Discussion

We assessed the prevalence of thirteen health-risk behaviours related to food consumption frequencies and physical activity among primary-school children in five European countries and examined their association, both individually and combined, with obesity and overweight. The highest prevalence of many risk behaviours was observed in Bulgaria and the lowest in Sweden, whereas the other three countries did not show a clear country order ranking pattern across the behaviours. For instance, Portugal was ranked just before Sweden for the food-related but not for the physical activity-related risk behaviours. Bulgarian and Portuguese children were more obese than Czech, Lithuanian and Swedish children. The statistically significant associations found between the food-related risk behaviours and obesity showed contrasting results although they were consistent across the countries, whereas the significant associations between physical activity-related risk behaviours and obesity were all positive. Moreover, the three calculated risk behaviour scores were positively associated with obesity, most pronounced in Swedish children or in the group of children in the five countries.

To our knowledge, nationally representative European-wide studies collecting data on behaviours related to both nutrition and/or physical activity, as well as using a common data collection protocol, are scarce. Two studies, both targeting adolescents, could be identified: the HBSC targeting 11-, 13- and 15-year-olds^(^ ^16^ ^)^ and the ‘European energy balance research to prevent excessive weight gain among youth (ENERGY)’ project targeting 10–12-year-olds^(^ ^28^ ^)^. We could compare our results with those for one behaviour only in each of these two studies. The other behavioural indicators that were given in their reports were defined differently or presented in another way. Comparing with the results from the 11-year-olds in the HBSC survey from 2005/2006^(^ ^16^ ^)^, slight differences in the risk behaviour ‘eating fruit <7 d/week’ are seen in HBSC (Portugal, 48 %; Bulgaria, 52 %; Czech Republic, 57 %, Sweden, 59 %; Lithuania, 72 %), whereby Bulgaria was grouped in the more favourable and Sweden in the less favourable country group, which is opposite to our data (Table 3)^(^ ^16^ ^)^. The percentage of children in COSI who did not have breakfast every day (range 4–33 %) was on average lower than the percentage found in 10–12-year-old adolescents in the seven European countries participating in the ENERGY project (boys, 17–52 %; girls, 12–51 %)^(^ ^28^ ^)^. This difference may be due to the younger age of the COSI children as it has been suggested elsewhere that skipping breakfast is more prevalent among older children^(^ ^29^ ^)^.

Other identified European-wide studies included sub-nationally representative samples of children^(^ ^30^ ^,^ ^31^ ^)^ or adolescents^(^ ^32^ ^)^ or secondary data sets based on different measures^(^ ^33^ ^)^. Therefore comparisons of their data with ours are difficult.

We assessed the average number of less favourable behaviours present by assigning a risk score to each child. We could identify two Dutch studies that also summarized the number of behaviours children were engaged in by means of a risk score^(^ ^34^ ^,^ ^35^ ^)^. However, comparisons with their results were not possible because of the use of different and fewer behavioural indicators.

None of the six abovementioned European-wide studies^(^ ^16^ ^,^ ^28^ ^,^ ^30^ ^–^ ^33^ ^)^ that have collected data on individual behaviours related to both nutrition and/or physical activity used the score approach. Instead, three^(^ ^36^ ^–^ ^38^ ^)^ have performed cluster analysis to study interrelationships among multiple behaviours and to identify high-risk groups of children in Europe, whereby country representation varied in the different clusters^(^ ^36^ ^)^ and not necessarily all less favourable behaviours occurred simultaneously in a cluster^(^ ^36^ ^–^ ^38^ ^)^. This has also been shown in country-specific studies that used similar food-related and/or physical activity-related behaviours^(^ ^39^ ^–^ ^41^ ^)^. COSI has been set up to monitor data by country and to make intercountry comparisons possible. We did not consider the available number of children with complete data on the thirteen risk behaviours sufficient to find a reasonable amount of differentiable clusters by country that would be large enough to warrant strategic attention^(^ ^42^ ^)^.

It is expected that children with excess body weight are more likely to be engaged in less favourable behaviours. This was the case in our study for two food-related risk behaviours: not having breakfast every day and eating ‘foods like pizza, French fries (chips), hamburgers, sausages or meat pies’ >3 d/week (Table 6). But we also found the opposite to be true for three food-related risk behaviours of not eating fruit every day, eating ‘foods like potato chips (crisps), corn chips, popcorn or peanuts’ >3 d/week and eating ’foods like biscuits, cakes, doughnuts or pies’ >3 d/week (Table 6), whereby children performing these three behaviours were less likely to be obese or overweight (Supplementary Table 5). A possible explanation for these results may be the cross-sectional nature of the COSI data and thus reverse causality has to be taken into account. Children with excess body weight may already have changed their eating patterns and therefore have truthfully answered the form according to the current situation and not according to what actually may have led to their overweight status.

Positive associations between skipping breakfast and excess weight have been also been reported by several other studies^(^ ^34^ ^,^ ^43^ ^,^ ^44^ ^)^, while to our knowledge a negative association between low fruit intake and obesity or overweight in just a few^(^ ^33^ ^)^. We did not find relevant studies to compare with for the other two less favourable food-related behaviours (items (v) and (vii)) for which we found statistically significant negative associations with obesity and overweight, and the second less favourable food-related behaviour (item (viii)) for which we found a statistically significant positive association with obesity. Concerning the five physical-activity related risk behaviours in our study, spending screen time ≥2 h/d and playing outside <1 h/d were the only behaviours that were statistically significantly associated with higher odds of obesity. Positive associations between watching television and other screen activities and BMI have also been reported by several other studies^(^ ^33^ ^,^ ^34^ ^,^ ^45^ ^)^.

It has been suggested that a combination of less favourable behaviours related to both nutrition and physical activity may have a possible synergetic effect that could lead to a multiplication of the risk of obesity or overweight^(^ ^34^ ^,^ ^36^ ^,^ ^38^ ^–^ ^40^ ^)^. In our study, we found for the total group of children in the five countries that a health-risk behaviour score of 3–8 or a score of 11 (thus a combination of three or more less favourable behaviours on both nutrition and physical activity), as well as a physical activity-risk behaviour score of 2–4, led to higher odds of obesity (Table 7). This was not shown for the food-risk behaviour score.

Several methodological issues of the present study need to be acknowledged. Its strengths include the availability of nationally representative samples of more than 10 000 children, the administration of the same COSI family form in five countries, which enabled the intercountry comparisons, as well as the standardized weight and height measurements^(^ ^12^ ^)^. However, the study also has some limitations.

The first set of concerns relates to the questionnaire used. For instance, the questions on the children’s behaviours were adapted from the HBSC 2002 questionnaire^(^ ^15^ ^)^ that has been validated among adolescents^(^ ^46^ ^)^, but to our knowledge not among children within the COSI targeted age range of 6–9 years. Furthermore, in the COSI questions we used fewer answer categories than incorporated in the HBSC questionnaire, because the COSI family form was designed to give a rough indication of the prevalence of the children’s health-risk behaviours and not for detailed analyses between risk behaviours and health outcomes. For instance, the COSI FFQ uses four frequency categories for a usual week while the 2001/2002 HBSC uses seven^(^ ^15^ ^)^. In our questionnaire, we also did not collect data on portion sizes, which have been suggested to be positively associated with obesity^(^ ^47^ ^)^.

A second set of concerns relates to filling in the questionnaire. For example, the data were reported by the parents (possibly together with their child). COSI was introduced to the parents as a European Childhood Growth Study with the aim to promote the health and well-being among primary-school children, the words overweight or obesity were not mentioned, and the children’s height and weight measurement values were only provided to the parents upon request. Nevertheless, it might be that parents were aware of their child’s weight status and that this could have influenced the report on their child’s behaviours. Parents with overweight children are likely to over-report more favourable behaviours and under-report less favourable behaviours^(^ ^48^ ^,^ ^49^ ^)^, and parents who are concerned about their child’s weight status are more likely to limit child screen time, take steps to improve their child’s diet or increase their child’s physical activity^(^ ^50^ ^)^. In addition, we do not know to what extent the parents completed the form solely by themselves or together with their child. A validation study comparing the children’s report with their parent’s report on the children’s energy intake using FFQ suggests that children (aged 8–11 years) are more accurate reporters than their parents, and that fathers are more accurate than mothers^(^ ^51^ ^)^.

A third concern relates to the representativeness of the children in our analysis. One-third of the children included in the analysis had a missing value on any of the health-risk behaviours or on children’s residential urbanization grade, parental education or parental occupation. Compared with the group of children with missing values (*n* 5126), the subgroup of children without any missing values (*n* 10 517) included 14·2 % fewer children whose parents had a low educational level, 2·3 % fewer children whose parents were both unemployed and 5·6 % more children who lived in the urban area. In addition, the subgroup contained fewer children for five less favourable behaviours (range absolute difference: –1 to –8 %) and more children for four less favourable behaviours (range absolute difference: +2 to +4 %). While these statistically significant, although relatively small, group differences may have influenced the results of the multivariable analyses, it is likely that the missing data were at random and that the effect estimates were not biased^(^ ^52^ ^)^.

A final limitation relates to the fact that only five out of thirteen countries administered the voluntary family form in COSI round 2007/2008. This allows us to make some intercountry comparisons, but obviously the number of countries is too small to identify groups of countries with similar patterns of health-risk behaviours like we could do with the data on the children’s weight and height measurements^(^ ^14^ ^)^ and with the data on the school nutrition environment^(^ ^53^ ^)^.

## Conclusion

In conclusion, despite a categorization of behaviours that was based on international health recommendations, only four out of thirteen health-risk behaviours were found to be positively associated with obesity and three were even found to be negatively associated with obesity or overweight. A combination of health-risk behaviours, on the other hand, showed more consistent findings and all in the same direction. The significant positive associations found between the physical activity-risk scores and obesity, as well as between the health-risk scores and obesity, underline the importance of, in particular, promoting physical activity-related and discouraging sedentary behaviours among schoolchildren in the context of obesity preventive interventions.

Given the strengths and limitations, the data collected in the present study can be considered valuable at the country level to indicate the level of behavioural health risks on nutrition and physical activity among primary-school children. The results show that with the present data it is possible to investigate variations in behaviours across countries, but to identify sub-European differences in behavioural health risks among schoolchildren, data from more countries should be collected. COSI includes repeated data collection rounds in 2−3-year intervals, whereby with each round more countries are expected to join^(^ ^12^ ^)^. It is thus envisaged that future rounds may provide more explanatory suggestions for the overweight north–south gradient found in other COSI analyses, whereby the highest prevalence was found in southern European countries^(^ ^14^ ^,^ ^54^ ^)^.

## Supplementary material

For supplementary material accompanying this paper visit http://dx.doi.org/10.1017/S1368980015001937. click here to view supplementary material

## References

[1] World Health Organization (2012) Population-Based Approaches to Childhood Obesity Prevention. Geneva: WHO; available at http://apps.who.int/iris/bitstream/10665/80149/1/9789241504782_eng.pdf

[2] Branca F, Nikogosian H & Lobstein T (editors) (2007) The Challenge of Obesity in the WHO European Region and the Strategies for Response. Copenhagen: WHO Regional Office for Europe; available at http://www.euro.who.int/__data/assets/pdf_file/0010/74746/E90711.pdf

[3] de Onis M, Blössner M & Borghi E (2010) Global prevalence and trends of overweight and obesity among preschool children. Am J Clin Nutr 92, 1257–1264. 20861173 10.3945/ajcn.2010.29786

[4] Muthuri SK, Francis CE, Wachira LJ et al (2014) Evidence of an overweight/obesity transition among school-aged children and youth in Sub-Saharan Africa: a systematic review. PLoS One 9, e92846. 24676350 10.1371/journal.pone.0092846 PMC3968060

[5] Mirmiran P, Sherafat-Kazemzadeh R, Jalali-Farahani S et al (2010) Childhood obesity in the Middle East: a review. East Mediterr Health J 16, 1009–1017. 21218730

[6] European Commission (2014) EU Action Plan on Childhood Obesity 2014−2020. Brussels: EC.

[7] World Health Organization (2014) Report of the First Meeting of the Ad hoc Working Group on Science and Evidence for Ending Childhood Obesity, 18–20 June 2014, Geneva, Switzerland. Geneva: WHO; available at http://www.who.int/end-childhood-obesity/echo-final-report-august-2014.pdf?

[8] Craigie AM, Lake AA, Kelly SA et al (2011) Tracking of obesity-related behaviours from childhood to adulthood: a systematic review. Maturitas 70, 266–284. 21920682 10.1016/j.maturitas.2011.08.005

[9] Singh AS, Mulder C, Twisk JW et al (2008) Tracking of childhood overweight into adulthood: a systematic review of the literature. Obes Rev 9, 474–488. 18331423 10.1111/j.1467-789X.2008.00475.x

[10] Wilkinson JR, Walrond S, Ells LJ et al (2007) Surveillance and monitoring. Obes Rev 8, Suppl. 1, 23–29. 17316297 10.1111/j.1467-789X.2007.00313.x

[11] World Health Organization Regional Office for Europe (2006) European Charter on Counteracting Obesity. Copenhagen: WHO Regional Office for Europe; available at http://www.euro.who.int/__data/assets/pdf_file/0009/87462/E89567.pdf

[12] Wijnhoven T, van Raaij J & Breda J (2014) WHO European Childhood Obesity Surveillance Initiative. Implementation of Round 1 (2007/2008) and Round 2 (2009/2010). Copenhagen: WHO Regional Office for Europe; available at http://www.euro.who.int/__data/assets/pdf_file/0004/258781/COSI-report-round-1-and-2_final-for-web.pdf?

[13] Wijnhoven T & Branca F (2008) WHO European Childhood Obesity Surveillance Initiative. Protocol, version January 2008. Copenhagen: WHO Regional Office for Europe.

[14] Wijnhoven TMA, van Raaij JMA, Spinelli A et al (2013) WHO European Childhood Obesity Surveillance Initiative 2008: weight, height and body mass index in 6–9-year-old children. Pediatr Obes 8, 79–97. 23001989 10.1111/j.2047-6310.2012.00090.x

[15] Currie C, Samdal O, Boyce W et al (editors) (2002) Health Behaviour in School-Aged Children: A WHO Cross-National Study. Research Protocol for the 2001/2002 Survey. Edinburgh: University of Edinburgh.

[16] Currie C, Gabhainn SN, Godeau E et al (editors) (2008) Inequalities in Young People’s Health. Health Behaviour in School-Aged Children International Report from the 2005/2006 Survey. Copenhagen: WHO Regional Office for Europe; available at http://www.euro.who.int/__data/assets/pdf_file/0005/53852/E91416.pdf

[17] World Health Organization (2009) Global School-Based Student Health Survey. Part 12: Core Module Rationale. Geneva: WHO; available at http://www.who.int/chp/gshs/GSHS_Item_Rationales_2009_English.pdf

[18] Centers for Disease Control and Prevention (2013) Nutrition for Everyone: Fruits and Vegetables. http://www.cdc.gov/nutrition/everyone/fruitsvegetables/index.html (accessed March 2015).

[19] World Health Organization Regional Office for Europe (2006) Food and Nutrition Policy for Schools. A Tool for the Development of School Nutrition Programmes in the European Region. Copenhagen: WHO Regional Office for Europe; available at http://www.euro.who.int/__data/assets/pdf_file/0019/152218/E89501.pdf?ua=1

[20] World Health Organization (2010) Set of Recommendations on the Marketing of Foods and Non-Alcoholic Beverages to Children. Geneva: WHO; available at http://whqlibdoc.who.int/publications/2010/9789241500210_eng.pdf

[21] World Health Organization (2010) Global Recommendations on Physical Activity for Health. Geneva: WHO; available at http://whqlibdoc.who.int/publications/2010/9789241599979_eng.pdf?ua=1 26180873

[22] American Academy of Pediatrics Committeee on Public Education (2001) Children, adolescents, and television. Pediatrics 107, 423–426. 11158483 10.1542/peds.107.2.423

[23] World Health Organization Regional Office for Europe (2004) *WHO Technical Meeting on Sleep and Health, Bonn, Germany, 22–24 January 2004* Copenhagen: WHO Regional Office for Europe; available at http://www.euro.who.int/__data/assets/pdf_file/0008/114101/E84683.pdf

[24] World Health Organization (1995) Physical Status: The Use and Interpretation of Anthropometry. Report of a WHO Expert Committee. WHO Technical Report Series, no. 854. Geneva: WHO; available at http://apps.who.int/iris/bitstream/10665/37003/1/WHO_TRS_854.pdf 8594834

[25] de Onis M, Onyango AW, Borghi E et al (2007) Development of a WHO growth reference for school-aged children and adolescents. Bull World Health Organ 85, 660–667. 18026621 10.2471/BLT.07.043497 PMC2636412

[26] Blössner M, Siyam A, Borghi E et al (2009) WHO AnthroPlus for Personal Computers Manual. Software for Assessing Growth of the World’s Children and Adolescents. Geneva: WHO; available at http://www.who.int/growthref/tools/who_anthroplus_manual.pdf

[27] Marascuilo LA (1966) Large-sample multiple comparison. Psychol Bull 65, 280–290. 5325892 10.1037/h0023189

[28] Brug J, van Stralen MM, te Velde SJ et al (2012) Differences in weight status and energy-balance related behaviours among schoolchildren across Europe: the ENERGY-project. PLoS One 7, e34742. 22558098 10.1371/journal.pone.0034742 PMC3338827

[29] Rampersaud GC, Pereira MA, Girard BL et al (2005) Breakfast habits, nutritional status, body weight, and academic performance in children and adolescents. J Am Diet Assoc 105, 743–760. 15883552 10.1016/j.jada.2005.02.007

[30] Riddoch C, Edwards D, Page A et al (2005) The European Youth Heart Study – cardiovascular disease risk factors in children: rationale, aims, study design and validation of methods. J Phys Act Health 2, 115–129.

[31] Ahrens W, Bammann K, de Henauw S et al (2006) Understanding and preventing childhood obesity and related disorders – IDEFICS: a European multilevel epidemiological approach. Nutr Metab Cardiovasc Dis 16, 302–308. 16679223 10.1016/j.numecd.2006.01.011

[32] Moreno LA, De Henauw S, González-Gross M et al (2008) Design and implementation of the Healthy Lifestyle in Europe by Nutrition in Adolescence Cross-Sectional Study. Int J Obes (Lond) 32, Suppl. 5, S4–S11. 19011652 10.1038/ijo.2008.177

[33] van Stralen MM, te Velde SJ, van Nassau F et al (2012) Weight status of European preschool children and associations with family demographics and energy balance-related behaviours: a pooled analysis of six European Studies. Obes Rev 13, Suppl. 1, 29–41. 22309063 10.1111/j.1467-789X.2011.00959.x

[34] Veldhuis L, Vogel I, Renders CM et al (2012) Behavioral risk factors for overweight in early childhood; the ‘Be active, eat right’ study. Int J Behav Nutr Phys Act 9, 74. 22704042 10.1186/1479-5868-9-74 PMC3409071

[35] Jansen W, Mackenbach JP, Joosten-van Zwanenburg E et al (2010) Weight status, energy-balance behaviours and intentions in 9–12-year-old inner-city children. J Hum Nutr Diet 23, 85–96. 20078731 10.1111/j.1365-277X.2009.01027.x

[36] Fernández-Alvira JM, De Bourdeaudhuij I, Singh AS et al (2013) Clustering of energy balance-related behaviors and parental education in European children: the ENERGY project. Int J Behav Nutr Phys Act 10, 5. 23320538 10.1186/1479-5868-10-5 PMC3618064

[37] Bel-Serrat S, Mouratidou T, Santaliestra-Pasías AM et al (2013) Clustering of multiple lifestyle behaviours and its association to cardiovascular risk factors in children: the IDEFICS study. Eur J Clin Nutr 67, 848–854. 23632753 10.1038/ejcn.2013.84

[38] Ottevaere C, Huybrechts I, Benser J et al (2011) Clustering patterns of physical activity, sedentary and dietary behavior among European adolescents: the HELENA study. BMC Public Health 11, 328. 21586158 10.1186/1471-2458-11-328 PMC3112135

[39] Landsberg B, Plachta-Danielzik S, Lange D et al (2010) Clustering of lifestyle factors and assocation with overweight in adolescents of the Kiel Obesity Prevention Study. Public Health Nutr 13, 1708–1715. 20883570 10.1017/S1368980010002260

[40] Sabbe D, De Bourdeaudhuij I, Legiest E et al (2008) A cluster-analytical approach towards physical activity and eating habits among 10-year-old children. Health Educ Res 23, 753–762. 18024978 10.1093/her/cyl135

[41] Cameron AJ, Crawford DA, Salmon J et al (2011) Clustering of obesity-related risk behaviors in children and their mothers. Ann Epidemiol 21, 95–102. 21184950 10.1016/j.annepidem.2010.11.001

[42] Mooi E & Sarstedt M (2011) Cluster analysis In A Concise Guide to Market Research: The Process, Data, and Methods Using IBM SPSS Statistics, 2nd ed, pp. 237–284 [E Mooi and M Sarstedt, editors]. Heidelberg: Springer-Verlag.

[43] Horikawa C, Kodama S, Yachi Y et al (2011) Skipping breakfast and prevalence of overweight and obesity in Asian and Pacific regions: a meta-analysis. Prev Med 53, 260–267. 21925535 10.1016/j.ypmed.2011.08.030

[44] Szajewska H & Ruszczynski M (2010) Systematic review demonstrating that breakfast consumption influences body weight outcomes in children and adolescents in Europe. Crit Rev Food Sci Nutr 50, 113–119. 20112153 10.1080/10408390903467514

[45] Olafsdottir S, Berg C, Eiben G et al (2014) Young children’s screen activities, sweet drink consumption and anthropometry: results from a prospective European study. Eur J Clin Nutr 68, 223–228. 24253759 10.1038/ejcn.2013.234

[46] Roberts C, Freeman J, Samdal O et al (2009) The Health Behaviour in School-aged Children (HBSC) study: methodological developments and current tensions. Int J Public Health 54, Suppl. 2, 140–150. 19639259 10.1007/s00038-009-5405-9 PMC2732766

[47] Albar SA, Alwan NA, Evans CE et al (2014) Is there an association between food portion size and BMI among British adolescents? Br J Nutr 112, 841–851. 24998364 10.1017/S0007114514001548

[48] Börnhorst C, Huybrechts I, Ahrens W et al (2013) Prevalence and determinants of misreporting among European children in proxy-reported 24 h dietary recalls. Br J Nutr 109, 1257–1265. 22863030 10.1017/S0007114512003194

[49] Fisher JO, Johnson RK, Lindquist C et al (2000) Influence of body composition on the accuracy of reported energy intake in children. Obes Res 8, 597–603. 11156436 10.1038/oby.2000.77

[50] Moore LC, Harris CV & Bradlyn AS (2012) Exploring the relationship between parental concern and the management of childhood obesity. Matern Child Health J 16, 902–908. 21594667 10.1007/s10995-011-0813-x

[51] Burrows TL, Truby H, Morgan PJ et al (2013) A comparison and validation of child versus parent reporting of children’s energy intake using food frequency questionnaires versus food records: who’s an accurate reporter? Clin Nutr 32, 613–618. 23206381 10.1016/j.clnu.2012.11.006

[52] Bhaskaran K & Smeeth L (2014) What is the difference between missing completely at random and missing at random? Int J Epidemiol 43, 1336–1339. 24706730 10.1093/ije/dyu080 PMC4121561

[53] Wijnhoven TMA, van Raaij JMA, Sjöberg A et al (2014) WHO European Childhood Obesity Surveillance Initiative: school nutrition environment and body mass index in primary schools. Int J Environ Res Public Health 11, 11261–11285. 25361044 10.3390/ijerph111111261 PMC4245612

[54] Wijnhoven TMA, van Raaij JMA, Spinelli A et al (2014) WHO European Childhood Obesity Surveillance Initiative: body mass index and level of overweight among 6–9-year-old children from school year 2007/2008 to school year 2009/2010. BMC Public Health 14, 806. 25099430 10.1186/1471-2458-14-806 PMC4289284

