# Supplementary material for: WHO European Childhood Obesity Surveillance Initiative: health-risk behaviours on nutrition and physical activity in 6–9-year-old schoolchildren
Source: Public Health Nutr. 2015 Jul 1;18(17):3108–24. doi: 10.1017/S1368980015001937 (PMC4642225; doi:10.1017/S1368980015001937)
Supplement: Supplementary file 1 [file S1368980015001937sup001.docx]

**Supplementary Table 1.** Characteristics of parental education, parental occupation and children’s residential urbanization grade of the total study group^*^, by country

| **Characteristics** | **Bulgaria** | **Czech Republic** | **Lithuania** | **Portugal** | **Sweden** |
| --- | --- | --- | --- | --- | --- |
|  | % | | | | |
| *Maternal education* (total *n*)^†^ | 3165 | 1587 | 3977 | 2931 | 3554 |
| Primary school | 23.8 | 9.2 | 10.5 | 23.7 | 5.9 |
| Secondary school | 43.9 | 64.5 | 38.9 | 59.8 | 53.1 |
| Undergraduate/bachelor’s degree | 12.3 | 8.4 | 40.3 | 14.4 | 34.1 |
| Master’s degree or higher | 20.1 | 18.0 | 10.4 | 2.1 | 7.0 |
| *Paternal education* (total *n*)^†^ | 3095 | 1549 | 3625 | 2869 | 3365 |
| Primary school | 22.8 | 9.6 | 10.8 | 29.3 | 9.4 |
| Secondary school | 55.5 | 62.6 | 46.5 | 59.2 | 59.0 |
| Undergraduate/bachelor’s degree | 7.0 | 7.7 | 34.8 | 9.9 | 23.6 |
| Master’s degree or higher | 14.6 | 20.1 | 7.9 | 1.6 | 7.9 |
| *Parental education* (total *n*)^†^ | 3062 | 1546 | 3592 | 2844 | 3322 |
| Both parents low^‡^ | 63.2 | 62.0 | 37.9 | 80.2 | 49.3 |
| *Maternal occupation* (total *n*)^†^ | 3159 | 1566 | 3974 | 2920 | 3353 |
| Government employed | 19.4 | 23.4 | 31.3 | 60.8^§^ | 39.8 |
| Non-government employed | 40.4 | 37.6 | 37.8 | – | 39.3 |
| Self-employed | 7.5 | 8.8 | 5.2 | 10.9 | 7.2 |
| Student | 0.7 | 0.1 | 2.1 | 0.8 | 6.2 |
| Homemaker | 19.2 | 25.7 | 18.6 | 18.4 | 2.5 |
| Unemployed, able to work | 11.8 | 3.8 | 3.3 | 7.9 | 2.0 |
| Unemployed, unable to work | 0.6 | 0.6 | 1.7 | 0.8 | 1.4 |
| Retired | 0.4 | 0.1 | 0.0 | 0.4 | 1.7 |
| *Paternal occupation* (total *n*)^†^ | 3042 | 1516 | 3551 | 2854 | 3228 |
| Government employed | 19.8 | 18.9 | 23.3 | 72.3^§^ | 18.4 |
| Non-government employed | 52.2 | 49.9 | 53.3 | – | 58.8 |
| Self-employed | 14.3 | 27.6 | 15.3 | 21.3 | 18.8 |
| Student | 0.1 | 0.0 | 0.2 | 0.0 | 1.2 |
| Homemaker | 0.3 | 0.3 | 1.3 | 0.3 | 0.2 |
| Unemployed, able to work | 11.6 | 2.2 | 5.0 | 4.2 | 0.9 |
| Unemployed, unable to work | 1.0 | 0.8 | 1.7 | 1.1 | 0.7 |
| Retired | 0.8 | 0.4 | 0.0 | 0.8 | 1.0 |
| *Parental occupation* (total *n*)^†^ | 3013 | 1497 | 3511 | 2806 | 3106 |
| Both parents unemployed | 10.4 | 2.3 | 3.6 | 3.0 | 2.0 |
| Children’s residential urbanization grade (total *n*)^†^ | 3267 | 1556 | 4084 | 3026 | 3633 |
| Urban | 78.8 | 47.8 | 32.8 | 66.4 | 29.2 |
| Semi-urban | 0.1 | 23.5 | 40.9 | 20.2 | 15.5 |
| Rural | 21.2 | 28.7 | 26.3 | 13.5 | 55.3 |

–, not applied as answer option; BMI, body mass index.

^*^ Children with complete information on sex, whose age was between 6 and 9 years old, whose weight and height were measured, whose BMI-for-age Z-score was within the normal range (≥–5 – ≤+5) and who returned a filled out family record form.

^†^ Statistically significant difference of proportions across the countries (chi-squared test; P<0.001).

^‡^ The highest educational level of both parents is either primary or secondary school.

^§^ This value included ‘government-employed’ and ‘non-government employed’.

**Supplementary Table 2.** Proportion (%) of consumption frequencies of breakfast and eight food items over a typical or usual week for the total study group^*^, by country

| **Consumption frequency** | **Bulgaria** | **Czech Republic** | **Lithuania** | **Portugal** | **Sweden** |
| --- | --- | --- | --- | --- | --- |
|  | % | | | | |
| Breakfast (total *n*)^†^ | 3246 | 1604 | 4036 | 3002 | 3626 |
| Every day | 78.9 | 75.7 | 67.5 | 95.6 | 94.4 |
| 4–6 days | 12.2 | 10.8 | 7.9 | 2.0 | 3.8 |
| 1–3 days | 7.5 | 11.2 | 14.3 | 1.8 | 1.6 |
| Never | 1.5 | 2.4 | 10.3 | 0.6 | 0.2 |
| Fresh fruit (total *n*)^†^ | 3202 | 1590 | 4008 | 2958 | 3608 |
| Every day | 27.1 | 52.0 | 31.4 | 61.5 | 66.7 |
| 4–6 days | 29.5 | 32.3 | 28.4 | 18.1 | 19.7 |
| 1–3 days | 41.5 | 14.8 | 39.4 | 18.4 | 12.0 |
| Never | 2.0 | 0.9 | 0.8 | 2.0 | 1.8 |
| 100% fruit juice (total *n*)^†^ | 3145 | 1562 | 3914 | 2856 | 3585 |
| Every day | 18.7 | 5.1 | 13.8 | 13.8 | 11.6 |
| 4–6 days | 19.5 | 9.2 | 19.8 | 13.3 | 13.8 |
| 1–3 days | 48.9 | 61.1 | 63.0 | 50.8 | 53.4 |
| Never | 13.0 | 24.6 | 3.4 | 22.1 | 21.3 |
| Vegetables (excluding potatoes) (total *n*)^†^ | 3174 | 1582 | 3992 | 2890 | 3599 |
| Every day | 23.6 | 28.3 | 23.3 | 39.5 | 53.4 |
| 4–6 days | 36.0 | 36.4 | 32.1 | 25.4 | 29.5 |
| 1–3 days | 38.2 | 33.3 | 42.7 | 31.6 | 15.4 |
| Never | 2.2 | 2.0 | 1.9 | 3.5 | 1.7 |
| Soft drinks containing sugar (total *n*)^†^ | 3190 | 1563 | 3955 | 2852 | 3597 |
| Every day | 21.9 | 25.3 | 9.4 | 9.9 | 1.5 |
| 4–6 days | 16.7 | 21.2 | 10.1 | 10.3 | 7.0 |
| 1–3 days | 40.8 | 39.8 | 65.7 | 46.5 | 78.5 |
| Never | 20.7 | 13.7 | 14.8 | 33.3 | 13.0 |
| Foods like potato chips (crisps), corn chips, popcorn or peanuts (total *n*)^†^ | 3224 | 1570 | 3989 | 2901 | 3608 |
| Every day | 21.3 | 1.4 | 3.0 | 1.6 | 0.1 |
| 4–6 days | 25.2 | 2.9 | 6.6 | 3.9 | 1.0 |
| 1–3 days | 49.8 | 69.0 | 80.8 | 60.9 | 83.7 |
| Never | 3.7 | 26.7 | 9.5 | 33.6 | 15.2 |
| Foods like candy bars or chocolate (total *n*)^†^ | 3237 | 1599 | 4010 | 2897 | 3616 |
| Every day | 33.6 | 6.3 | 18.3 | 2.7 | 0.3 |
| 4–6 days | 29.8 | 18.1 | 25.7 | 9.0 | 2.0 |
| 1–3 days | 35.3 | 71.0 | 55.6 | 73.2 | 93.0 |
| Never | 1.4 | 4.6 | 0.5 | 15.1 | 4.8 |
| Foods like biscuits, cakes, doughnuts or pies (total *n*)^†^ | 3218 | 1593 | 3929 | 2929 | 3608 |
| Every day | 20.5 | 5.6 | 12.6 | 8.4 | 0.6 |
| 4–6 days | 25.9 | 18.6 | 20.7 | 17.3 | 5.8 |
| 1–3 days | 49.2 | 71.6 | 65.6 | 65.6 | 83.3 |
| Never | 4.4 | 4.2 | 1.1 | 8.6 | 10.4 |
| Foods like pizza, French fries (chips), hamburgers, sausages or meat pies (total *n*)^†^ | 3238 | 1578 | 3982 | 2864 | 3615 |
| Every day | 17.1 | 0.5 | 1.6 | 1.3 | 0.2 |
| 4–6 days | 22.3 | 1.7 | 5.8 | 5.3 | 1.8 |
| 1–3 days | 56.7 | 68.0 | 81.2 | 72.4 | 90.4 |
| Never | 4.0 | 29.9 | 11.5 | 21.0 | 7.6 |

BMI, body mass index.

^*^ Children with complete information on sex, whose age was between 6 and 9 years old, whose weight and height were measured, whose BMI-for-age Z-score was within the normal range (≥–5 – ≤+5) and who returned a filled out family record form.

^†^ Statistically significant difference of proportions of the four answer options across the countries (chi-squared test; P<0.001).

**Supplementary Table 3.** Proportion (%) of items related to physical activity and screen time for the total study group^*^, by country

| **Items** | **Bulgaria** | **Czech Republic** | **Lithuania** | **Portugal** | **Sweden** |
| --- | --- | --- | --- | --- | --- |
|  | % | | | | |
| **Physical activity** |  |  |  |  |  |
| Usual transport going to school (total *n*)^†^ | 3255 | 1581 | 4069 | 2876 | 3609 |
| School bus | 8.2 | 5.6 | 7.3 | 15.8 | 9.3 |
| Public transport | 3.5 | 7.0 | 5.9 | 2.2 | 1.0 |
| Car | 28.6 | 32.5 | 38.3 | 56.2 | 39.5 |
| Bicycle | 0.9 | 1.3 | 0.3 | 0.3 | 9.6 |
| Walking | 58.7 | 52.1 | 48.0 | 25.0 | 32.7 |
| Other^‡^ | 0.0 | 1.6 | 0.2 | 0.5 | 7.9 |
| Usual transport coming from school (total *n*)^†^ | 3230 | 1522 | 4006 | 2816 | 3554 |
| School bus | 8.1 | 4.1 | 7.1 | 19.6 | 7.0 |
| Public transport | 4.1 | 7.0 | 7.9 | 1.9 | 1.0 |
| Car | 18.1 | 28.2 | 24.8 | 46.8 | 39.9 |
| Bicycle | 0.8 | 1.1 | 0.2 | 0.3 | 9.7 |
| Walking | 68.9 | 57.5 | 60.0 | 30.9 | 34.2 |
| Other^‡^ | 0.0 | 2.2 | 0.1 | 0.5 | 8.2 |
| Membership sport or dancing club (total *n*)^†^ | 3216 | 1561 | 3943 | 2939 | 3608 |
| Yes | 25.4 | 59.7 | 43.2 | 41.2 | 77.5 |
| Frequency sport or dancing club per week (total *n*)^†,§^ | 793 | 929 | 1703 | 1200 | 2797 |
| 0 days | 0.0 | 0.7 | 0.0 | 0.3 | 0.0 |
| 1 day | 15.5 | 39.4 | 29.1 | 27.8 | 34.0 |
| 2 days | 53.7 | 36.7 | 42.4 | 50.8 | 38.2 |
| 3 days | 20.2 | 16.5 | 20.1 | 14.4 | 20.5 |
| 4 days | 5.2 | 5.1 | 5.8 | 4.9 | 5.7 |
| 5 days | 4.7 | 1.4 | 2.4 | 1.5 | 1.4 |
| 6 days | 0.3 | 0.1 | 0.2 | 0.2 | 0.3 |
| 7 days | 0.5 | 0.2 | 0.1 | 0.1 | 0.0 |
| Playing outside on a weekday (total *n*)^†^ | 3236 | 1611 | 4040 | 2950 | 3610 |
| Never | 1.3 | 1.2 | 3.1 | 19.1 | 0.4 |
| <1 hour per day | 5.8 | 5.8 | 6.4 | 23.9 | 8.2 |
| About 1 hour per day | 19.8 | 28.4 | 20.4 | 28.5 | 25.9 |
| About 2 hours per day | 42.7 | 43.0 | 37.2 | 20.8 | 39.7 |
| About 3 or more hours per day | 30.4 | 21.7 | 32.9 | 7.7 | 25.8 |
| Playing outside on a weekend day (total *n*)^†^ | 3215 | 1589 | 3958 | 2908 | 3564 |
| Never | 0.4 | 0.1 | 0.5 | 6.6 | 0.1 |
| <1 hour per day | 1.1 | 0.4 | 0.7 | 5.6 | 1.7 |
| About 1 hour per day | 1.4 | 3.0 | 3.9 | 12.7 | 8.3 |
| About 2 hours per day | 14.6 | 17.7 | 12.0 | 24.0 | 28.3 |
| About 3 or more hours per day | 82.5 | 78.9 | 82.9 | 51.0 | 61.6 |
| **Screen time** |  |  |  |  |  |
| Using a computer on a weekday (total *n*)^†^ | 3008 | 1566 | 4024 | 2676 | 3613 |
| Never | 38.0 | 41.6 | 33.7 | 49.6 | 23.5 |
| <1 hour per day | 26.5 | 34.5 | 19.2 | 38.0 | 54.8 |
| About 1 hour per day | 24.2 | 19.6 | 31.7 | 9.9 | 17.9 |
| About 2 hours per day | 9.4 | 4.0 | 12.7 | 2.2 | 3.6 |
| About 3 or more hours per day | 2.0 | 0.3 | 2.7 | 0.3 | 0.3 |
| Using a computer on a weekend day (total *n*)^†^ | 3001 | 1556 | 3951 | 2672 | 3603 |
| Never | 33.4 | 27.8 | 25.8 | 28.7 | 11.6 |
| <1 hour per day | 19.2 | 35.5 | 12.8 | 32.5 | 43.8 |
| About 1 hour per day | 11.8 | 21.6 | 26.7 | 21.6 | 27.0 |
| About 2 hours per day | 26.2 | 12.9 | 24.8 | 13.5 | 13.9 |
| About 3 or more hours per day | 9.4 | 2.1 | 9.9 | 3.7 | 3.7 |
| Watching television on a weekday (total *n*)^†^ | 3248 | 1605 | 4071 | 2985 | 3616 |
| Never | 1.5 | 4.2 | 1.3 | 3.5 | 1.5 |
| <1 hour per day | 13.7 | 35.6 | 7.8 | 36.6 | 26.1 |
| About 1 hour per day | 30.6 | 40.8 | 35.6 | 34.6 | 49.5 |
| About 2 hours per day | 37.3 | 17.3 | 40.7 | 21.4 | 20.6 |
| About 3 or more hours per day | 16.8 | 2.1 | 14.6 | 4.1 | 2.3 |
| Watching television on a weekend day (total *n*)^†^ | 3235 | 1600 | 4010 | 2965 | 3606 |
| Never | 1.7 | 1.6 | 1.3 | 0.7 | 0.2 |
| <1 hour per day | 6.4 | 10.4 | 2.8 | 8.3 | 5.6 |
| About 1 hour per day | 6.4 | 27.8 | 15.5 | 21.1 | 20.2 |
| About 2 hours per day | 39.3 | 46.4 | 39.7 | 40.8 | 52.9 |
| About 3 or more hours per day | 46.2 | 13.8 | 40.8 | 29.0 | 21.1 |

BMI, body mass index.

^*^ Children with complete information on sex, whose age was between 6 and 9 years old, whose weight and height were measured, whose BMI-for-age Z-score was within the normal range (≥–5 – ≤+5) and who returned a filled out family record form.

^†^ Statistically significant difference of proportions of the answer options across the countries (chi-squared test; P<0.001).

^‡^ Other options for transport written down on the form were taxi, motorcycle, kick scooter or a combination of the listed options.

^§^ This question was only answered by the children who were members of a sport or dancing club.

**Supplementary Table 4.** Bivariate associations^†^ between 13 health-risk behaviours and overweight in the total study group^‡^, by country

|  | **Health-risk behaviour** | **Bulgaria** | **Czech Republic** | | | **Lithuania** | | **Portugal** | | **Sweden** | | **Total five countries** |
| --- | --- | --- | --- | --- | --- | --- | --- | --- | --- | --- | --- | --- |
|  |  | OR (95% CI) | | | | | | | | | | |
|  | *Breakfast and food consumption frequency* |  | |  |  | |  | |  | |  | |
| 1^§^ | Having breakfast <7 days per week | 1.11 (0.91;1.34) | **1.56 (1.19;2.04)***** | | | **1.39 (1.19;1.62)***** | | 1.38 (0.96;1.98) | | 1.25 (0.91;1.73) | | **1.31 (1.18;1.45)***** |
| 2^§^ | Eating fruit^ǁ^ <7 days per week | 0.93 (0.79;1.10) | 1.05 (0.83;1.34) | | | **0.83 (0.72;0.97)*** | | 0.97 (0.83;1.14) | | 0.94 (0.80;1.12) | | 0.93 (0.86;1.00) |
| 3^§^ | Eating vegetables (excluding potatoes) <7 days per week | **0.74 (0.61;0.89)***** | 1.08 (0.82;1.42) | | | 1.00 (0.84;1.19) | | 0.98 (0.83;1.15) | | 1.14 (0.97;1.33) | | 0.98 (0.90;1.06) |
| 4^§^ | Drinking soft drinks containing sugar >3 days per week | 0.92 (0.78;1.09) | 0.91 (0.71;1.17) | | | 0.97 (0.81;1.17) | | **1.23 (1.01;1.48)*** | | 1.16 (0.88;1.52) | | 1.00 (0.91;1.10) |
| 5^§^ | Eating foods like potato chips (crisps), corn chips, popcorn or peanuts >3 days per week | **0.79 (0.67;0.92)**** | 1.01 (0.55;1.86) | | | **0.72 (0.55;0.94)*** | | 0.90 (0.64;1.28) | | 0.49 (0.19;1.26) | | **0.79 (0.70;0.89)***** |
| 6^§^ | Eating foods like candy bars or chocolate >3 days per week | 0.97 (0.82;1.14) | **0.73 (0.54;0.99)*** | | | 0.90 (0.77;1.04) | | 0.94 (0.74;1.19) | | 1.06 (0.63;1.79) | | **0.91 (0.83;1.00)*** |
| 7^§^ | Eating foods like biscuits, cakes, doughnuts or pies >3 days per week | **0.74 (0.63;0.87)***** | **0.58 (0.42;0.79)***** | | | **0.81 (0.69;0.95)**** | | 0.86 (0.72;1.03) | | 0.84 (0.60;1.17) | | **0.78 (0.72;0.85)***** |
| 8^¶^ | Eating foods like pizza, French fries (chips), hamburgers, sausages or meat pies >3 days per week | 0.85 (0.72;1.01) | 1.40 (0.64;3.04) | | | 0.96 (0.72;1.29) | | 0.87 (0.63;1.19) | | 1.25 (0.74;2.13) | | 0.89 (0.79;1.01) |
|  | *Physical activity* |  |  | | |  | |  | |  | |  |
| 9^§^ | Using inactive transportation going to and from school | 1.07 (0.88;1.28) | 0.95 (0.73;1.24) | | | 1.13 (0.96;1.33) | | 1.02 (0.84;1.24) | | 1.10 (0.92;1.32) | | 1.07 (0.99;1.17) |
| 10^§^ | Going to a sport or dancing club <2 days per week | 0.93 (0.76;1.13) | 0.85 (0.66;1.09) | | | 1.15 (0.97;1.36) | | 1.01 (0.85;1.20) | | 1.08 (0.91;1.27) | | 1.02 (0.94;1.11) |
| 11^§^ | Playing outside <1 hour per day | 1.35 (0.95;1.93) | **0.51 (0.26;0.99)*** | | | 1.27 (0.97;1.67) | | 1.02 (0.86;1.20) | | 1.11 (0.83;1.49) | | 1.08 (0.96;1.22) |
|  | *Screen time and sleep duration* |  |  | | |  | |  | |  | |  |
| 12^§^ | Spending screen time ≥2 hours per day | **1.23 (1.02;1.49)*** | **1.52 (1.18;1.97)***** | | | **1.29 (1.08;1.54)**** | | **1.21 (1.02;1.44)*** | | **1.28 (1.09;1.50)**** | | **1.28 (1.18;1.38)***** |
| 13^§^ | Sleep duration <9 hours per day | 0.88 (0.71;1.08) | 1.09 (0.65;1.82) | | | 1.05 (0.83;1.31) | | 1.08 (0.86;1.35) | | **1.67 (1.12;2.50)*** | | 1.04 (0.92;1.17) |

BMI, body mass index; BMI/A, BMI-for-age; CI, confidence interval; OR, odds ratio; WHO, World Health Organization.

Significance levels: * *P*<0.05 ** *P*<0.01 *** *P*<0.001, significant associations are shown in bold.

^†^ All bivariate analyses were adjusted for the children’s sex and age and included random effects for the primary sampling units. The analyses for the five countries together also included random effects for country.

^‡^ Normal weight or overweight children with complete information on sex, whose age was between 6 and 9 years old, whose weight and height were measured, whose BMI/A Z-score was within the normal range (≥–5 – ≤+5) and who returned a filled out family record form. Overweight is defined as the proportion of children with a BMI/A value above +1 Z-score relative to the 2007 WHO growth reference median^(25)^ and was compared against normal weight children (BMI/A value ≥–2 Z-scores and ≤+1 Z-score).

^§^ Reference categories for each health-risk behaviour were: 1. having breakfast every day, 2. eating fruit every day, 3. eating vegetables (excluding potatoes) every day, 4. drinking soft drinks containing sugar ≤3 days per week, 5. eating foods like potato chips (crisps), corn chips, popcorn or peanuts ≤3 days per week, 6. eating foods like candy bars or chocolate ≤3 days per week, 7. eating foods like biscuits, cakes, doughnuts or pies ≤3 days per week, 8. eating foods like pizza, French fries (chips), hamburgers, sausages or meat pies ≤3 days per week, 9. using active transportation going to and from school, 10. going to a sport or dancing club ≥2 days per week, 11. playing outside ≥1 hour per day, 12. spending screen time <2 hours per day and 13. sleep duration ≥9 hours per day.

^ǁ^ Combination of ‘fresh fruit’ and ‘100% fruit juice’.

**Supplementary Table 5.** Multivariable associations^†^ between 13 health-risk behaviours and overweight in a subgroup of children without missing data^‡^, by country

|  | **Health-risk behaviour** | **Bulgaria** (*n*=2311) | **Czech Republic** (*n*=1077) | **Lithuania** (*n*=2817) | **Portugal** (*n*=1805) | **Sweden** (*n*=2315) | **Total five countries** (*n*=10 325) |
| --- | --- | --- | --- | --- | --- | --- | --- |
|  |  | OR (95% CI) | | | | | |
|  | *Breakfast and food consumption frequency* |  |  |  |  |  |  |
| 1^§^ | Having breakfast <7 days per week | **1.28 (1.01;1.61)*** | **1.53 (1.08;2.18)*** | **1.43 (1.19;1.72)***** | 1.55 (0.97;2.46) | 1.46 (0.94;2.27) | **1.39 (1.23;1.57)***** |
| 2^§^ | Eating fruit^ǁ^ <7 days per week | 0.98 (0.80;1.21) | 1.15 (0.81;1.62) | **0.80 (0.66;0.97)*** | 0.87 (0.70;1.08) | 0.89 (0.71;1.12) | **0.90 (0.81;0.99)*** |
| 3^§^ | Eating vegetables (excluding potatoes) <7 days per week | **0.75 (0.59;0.94)*** | 0.96 (0.65;1.42) | 1.04 (0.83;1.30) | 1.02 (0.83;1.26) | 1.12 (0.91;1.38) | 1.00 (0.90;1.11) |
| 4^§^ | Drinking soft drinks containing sugar >3 days per week | 1.09 (0.88;1.34) | 0.84 (0.61;1.15) | 1.10 (0.87;1.40) | **1.36 (1.05;1.76)*** | 1.01 (0.70;1.48) | 1.08 (0.96;1.21) |
| 5^§^ | Eating foods like potato chips (crisps), corn chips, popcorn or peanuts >3 days per week | 0.84 (0.68;1.03) | 1.22 (0.53;2.81) | **0.67 (0.45;0.98)*** | 1.24 (0.67;2.31) | 0.48 (0.15;1.60) | **0.81 (0.69;0.96)*** |
| 6^§^ | Eating foods like candy bars or chocolate >3 days per week | 1.05 (0.85;1.30) | 0.81 (0.54;1.21) | 1.03 (0.85;1.25) | 0.72 (0.50;1.05) | 1.15 (0.54;2.46) | 0.98 (0.87;1.10) |
| 7^§^ | Eating foods like biscuits, cakes, doughnuts or pies >3 days per week | **0.76 (0.61;0.95)*** | **0.61 (0.40;0.93)*** | **0.79 (0.64;0.97)*** | 0.87 (0.68;1.11) | 0.99 (0.65;1.51) | **0.80 (0.72;0.90)***** |
| 8^§^ | Eating foods like pizza, French fries (chips), hamburgers, sausages or meat pies >3 days per week | 0.99 (0.79;1.24) | 1.25 (0.43;3.67) | 1.42 (0.97;2.10) | 1.18 (0.68;2.05) | 1.54 (0.74;3.22) | 1.08 (0.91;1.28) |
|  | *Physical activity* |  |  |  |  |  |  |
| 9^§^ | Using inactive transportation going to and from school | 1.12 (0.90;1.38) | 0.85 (0.61;1.18) | 1.08 (0.89;1.31) | 1.03 (0.80;1.32) | 1.02 (0.82;1.26) | 1.06 (0.96;1.18) |
| 10^§^ | Going to a sport or dancing club <2 days per week | 1.10 (0.87;1.38) | 0.80 (0.58;1.10) | **1.25 (1.03;1.52)*** | 1.05 (0.84;1.31) | 0.99 (0.81;1.22) | 1.06 (0.96;1.17) |
| 11^§^ | Playing outside <1 hour per day | 1.26 (0.83;1.91) | 0.46 (0.19;1.10) | 1.28 (0.94;1.73) | 0.99 (0.81;1.22) | 0.84 (0.56;1.28) | 1.03 (0.90;1.19) |
|  | *Screen time and sleep duration* |  |  |  |  |  |  |
| 12^§^ | Spending screen time ≥2 hours per day | **1.29 (1.04;1.59)*** | **1.64 (1.18;2.27)**** | **1.33 (1.08;1.64)**** | **1.26 (1.02;1.56)*** | **1.25 (1.01;1.53)*** | **1.33 (1.20;1.46)***** |
| 13^§^ | Sleep duration <9 hours per day | 0.99 (0.78;1.26) | 0.66 (0.32;1.34) | 0.97 (0.74;1.27) | 0.90 (0.67;1.22) | 1.58 (0.94;2.65) | 1.00 (0.86;1.15) |

BMI, body mass index; BMI/A, BMI-for-age; CI, confidence interval; OR, odds ratio; WHO, World Health Organization.

Significance levels: * *P*<0.05 ** *P*<0.01 *** *P*<0.001, significant associations are shown in bold.

^†^ All multivariable analyses were adjusted for the children’s sex and age, included all 13 health-risk behaviours simultaneously, as well as children’s residential urbanization grade, parental education and parental occupation and included random effects for the primary sampling units. The analyses for the five countries together also included random effects for country.

^‡^ Normal weight or overweight children with complete information on sex, whose age was between 6 and 9 years old, whose weight and height were measured, whose BMI/A Z-score was within the normal range (≥–5 – ≤+5), who returned a filled out family record form and who had no missing values on any of the 13 health-risk behaviours, children’s residential urbanization grade, parental education and parental occupation. Overweight is defined as the proportion of children with a BMI/A value above +1 Z-score relative to the 2007 WHO growth reference median^(25)^ and was compared against normal weight children (BMI/A value ≥–2 Z-scores and ≤+1 Z-score).

^§^ Reference categories for each health-risk behaviour were: 1. having breakfast every day, 2. eating fruit every day, 3. eating vegetables (excluding potatoes) every day, 4. drinking soft drinks containing sugar ≤3 days per week, 5. eating foods like potato chips (crisps), corn chips, popcorn or peanuts ≤3 days per week, 6. eating foods like candy bars or chocolate ≤3 days per week, 7. eating foods like biscuits, cakes, doughnuts or pies ≤3 days per week, 8. eating foods like pizza, French fries (chips), hamburgers, sausages or meat pies ≤3 days per week, 9. using active transportation going to and from school, 10. going to a sport or dancing club ≥2 days per week, 11. playing outside ≥1 hour per day, 12. spending screen time <2 hours per day and 13. sleep duration ≥9 hours per day.

^ǁ^ Combination of ‘fresh fruit’ and ‘100% fruit juice’.

**Supplementary Table 6.** Associations^†^ between three risk behaviour scores and overweight in a subgroup of children without missing data^‡^, by country

| **Score categories** | **Bulgaria** | | **Czech Republic** | | **Lithuania** | | **Portugal** | | **Sweden** | | **Total five countries** | |
| --- | --- | --- | --- | --- | --- | --- | --- | --- | --- | --- | --- | --- |
|  | *n* | OR (95% CI) | *n* | OR (95% CI) | *n* | OR (95% CI) | *n* | OR (95% CI) | *n* | OR (95% CI) | *n* | OR (95% CI) |
| *Food-risk behaviour score* |  |  |  |  |  |  |  |  |  |  |  |  |
| 0 | 67 | 1.00 | 77 | 1.00 | 100 | 1.00 | 328 | 1.00 | 907 | 1.00 | 1479 | 1.00 |
| 1 | 136 | 0.71 (0.38;1.33) | 209 | 0.78 (0.42;1.45) | 350 | 1.06 (0.63;1.78) | 580 | 0.93 (0.70;1.24) | 750 | 1.20 (0.95;1.51) | 2025 | 1.06 (0.90;1.24) |
| 2 | 353 | 0.84 (0.48;1.46) | 304 | 0.70 (0.38;1.26) | 800 | 0.90 (0.56;1.47) | 537 | 1.03 (0.77;1.38) | 478 | 1.12 (0.86;1.47) | 2472 | 1.04 (0.88;1.21) |
| 3 | 436 | 0.84 (0.49;1.45) | 266 | 0.67 (0.37;1.24) | 807 | 1.14 (0.70;1.85) | 209 | 1.12 (0.78;1.62) | 125 | 1.06 (0.67;1.67) | 1843 | 1.12 (0.94;1.34) |
| 4 | 439 | 0.77 (0.45;1.34) | 145 | 0.62 (0.32;1.23) | 429 | 1.09 (0.66;1.81) | 97 | 1.14 (0.71;1.83) | 37 | 1.56 (0.75;3.25) | 1147 | 1.07 (0.88;1.31) |
| 5 | 421 | 0.73 (0.42;1.27) | 54 | 0.80 (0.35;1.85) | 224 | 0.87 (0.50;1.52) | 34 | 0.78 (0.36;1.68) | 11 | 3.21 (0.95;10.82) | 744 | 0.96 (0.76;1.20) |
| 6 | 297 | 0.64 (0.36;1.15) | 16 | 0.88 (0.25;3.12) | 82 | 1,28 (0.66;2.49) | 16 | 0.70 (0.23;2.12) | 4 | – | 415 | 0.89 (0.68;1.18) |
| 7 | 140 | 0.67 (0.35;1.27) | 5 | 1.52 (0.22;10.24) | 25 | 0.59 (0.18;1.91) | 4 | 1.72 (0.23;12.88) | 2 | – | 176 | 0.84 (0.58;1.24) |
| 8 | 22 | 0.40 (0.12;1.37) | 1 | – | 0 | – | 0 | – | 1 | – | 24 | 0.64 (0.23;1.76) |
| *Physical activity-risk behaviour score* |  |  |  |  |  |  |  |  |  |  |  |  |
| 0 | 88 | 1.00 | 154 | 1.00 | 116 | 1.00° | 53 | 1.00 | 407 | 1.00 | 818 | 1.00°° |
| 1 | 487 | 0.68 (0.41;1.12) | 448 | 0.82 (0.52;1.29) | 654 | 1.50 (0.89;2.54) | 402 | 1.08 (0.59;1.99) | 912 | **1.49 (1.10;2.03)*** | 2903 | 1.19 (0.98;1.44) |
| 2 | 1128 | 0.75 (0.46;1.20) | 352 | 1.03 (0.65;1.64) | 1206 | **1.80 (1.08;3.00)*** | 679 | 1.04 (0.57;1.88) | 739 | **1.62 (1.18;2.22)**** | 4104 | **1.30 (1.07;1.57)**** |
| 3 | 523 | 1.03 (0.63;1.68) | 106 | 0.82 (0.44;1.53) | 726 | **1.94 (1.15;3.27)*** | 496 | 1.24 (0.68;2.27) | 235 | 1.25 (0.82;1.90) | 2086 | **1.46 (1.19;1.79)***** |
| 4 | 78 | 0.97 (0.50;1.89) | 17 | 0.76 (0.20;2.82) | 102 | **2.26 (1.18;4.34)*** | 160 | 1.17 (0.61;2.27) | 22 | **2.73 (1.09;6.89)*** | 379 | **1.55 (1.17;2.05)**** |
| 5 | 7 | – | 0 | – | 13 | 2.14 (0.59;7.70) | 15 | 0.87 (0.25;2.97) | 0 | – | 35 | 0.95 (0.43;2.10) |
| *Health-risk behaviour score*^§^ |  |  |  |  |  |  |  |  |  |  |  |  |
| 0–1 | 37 | 1.00 | 84 | 1.00 | 51 | 1.00 | 134 | 1.00 | 674 | 1.00 | 980 | 1.00 |
| 2 | 102 | 1.10 (0.48;2.50) | 159 | 0.59 (0.31;1.09) | 192 | 1.55 (0.67;3.55) | 269 | 1.00 (0.64;1.55) | 631 | 1.20 (0.92;1.57) | 1353 | 1.11 (0.91;1.35) |
| 3 | 170 | 1.17 (0.54;2.54) | 243 | 0.53 (0.29;0.94) | 406 | 1.90 (0.87;4.19) | 411 | 1.02 (0.68;1.55) | 548 | 1.19 (0.90;1.58) | 1778 | 1.17 (0.97;1.42) |
| 4 | 303 | 1.03 (0.49;2.19) | 232 | 0.58 (0.32;1.03) | 594 | 1.72 (0.79;3.75) | 417 | 1.24 (0.82;1.87) | 286 | 1.35 (0.97;1.88) | 1832 | **1.22 (1.00;1.49)*** |
| 5 | 373 | 1.00 (0.48;2.11) | 175 | 0.56 (0.31;1.04) | 611 | 2.03 (0.93;4.42) | 292 | 1.19 (0.77;1.84) | 115 | 0.93 (0.56;1.53) | 1566 | 1.23 (1.00;1.50) |
| 6 | 361 | 0.93 (0.44;1.97) | 109 | 0.44 (0.22;0.89) | 495 | 1.79 (0.81;3.92) | 141 | 1.39 (0.84;2.29) | 41 | 1.74 (0.87;3.49) | 1147 | 1.17 (0.94;1.46) |
| 7 | 415 | 0.96 (0.46;2.02) | 43 | 0.92 (0.40;2.10) | 277 | 2.04 (0.91;4.58) | 93 | 1.02 (0.58;1.79) | 12 | 3.01 (0.92;9.81) | 840 | 1.22 (0.96;1.54) |
| 8 | 306 | 0.98 (0.46;2.08) | 21 | 0.53 (0.16;1.77) | 134 | 1.78 (0.75;4.19) | 32 | 1.12 (0.49;2.53) | 6 | 1.83 (0.32;10.43) | 499 | 1.13 (0.86;1.48) |
| 9 | 179 | 0.98 (0.44;2.16) | 8 | 2.07 (0.47;9.14) | 40 | **3.14 (1.15;8.56)*** | 11 | 0.17 (0.02;1.38) | 1 | – | 239 | 1.19 (0.84;1.67) |
| 10 | 58 | 1.16 (0.47;2.89) | 2 | – | 14 | 2.29 (0.57;9.26) | 4 | 1.84 (0.24;14.07) | 1 | – | 79 | 1.32 (0.78;2.21) |
| 11 | 6 | 0.37 (0.04;3.71) | 1 | – | 3 | 9.75 (0.78;122.42) | 1 | – | 0 | – | 11 | 2.29 (0.68;7.74) |
| 12 | 1 | – | 0 | – | 0 | – | 0 | – | 0 | – | 1 | – |
| 13 | 0 | – | 0 | – | 0 | – | 0 | – | 0 | – | 0 | – |

–, sample size was 0 or none of the children in this score category were overweight and thus the OR could not be estimated for this category; BMI, body mass index; BMI/A, BMI-for-age; CI, confidence interval; OR, odds ratio; WHO, World Health Organization.

Significance ORs: * *P*<0.05 ** *P*<0.01 *** *P*<0.001, significant associations are shown in bold; significant linear trend of ORs for the respective risk-behaviour score (likelihood ratio test): ° *P*<0.01 °° *P*<0.001.

^†^ All analyses were adjusted for the children’s sex and age, children’s residential urbanization grade, parental education and parental occupation and included random effects for the primary sampling units. The analyses for the five countries together also included random effects for country.

^‡^ Normal weight or overweight children with complete information on sex, whose age was between 6 and 9 years old, whose weight and height were measured, whose BMI/A Z-score was within the normal range (≥–5 – ≤+5), who returned a filled out family record form and who had no missing values on any of the 13 health-risk behaviours, children’s residential urbanization grade, parental education and parental occupation. Overweight is defined as the proportion of children with a BMI/A value above +1 Z-score relative to the 2007 WHO growth reference median^(25)^ and was compared against normal weight children (BMI/A value ≥–2 Z-scores and ≤+1 Z-score).

^§^ The reference category was not set as a health risk score of 0 but 0–1, because only 8 Bulgarian, 18 Czech, 7 Lithuanian, 10 Portuguese and 183 Swedish children obtained a health risk score of 0.
